# A human TRPV1 genetic variant within the channel gating domain regulates pain sensitivity in rodents

**DOI:** 10.1172/JCI163735

**Published:** 2023-02-01

**Authors:** Shufang He, Vanessa O. Zambelli, Pritam Sinharoy, Laura Brabenec, Yang Bian, Freeborn Rwere, Rafaela C.R. Hell, Beatriz Stein Neto, Barbara Hung, Xuan Yu, Meng Zhao, Zhaofei Luo, Chao Wu, Lijun Xu, Katrin J. Svensson, Stacy L. McAllister, Creed M. Stary, Nana-Maria Wagner, Ye Zhang, Eric R. Gross

**Affiliations:** 1 Department of Anesthesiology and Perioperative Medicine, the Second Hospital of Anhui Medical University, Key Laboratory of Anesthesiology and Perioperative Medicine of Anhui Higher Education Institutes, Anhui Medical University, Hefei, China.; 2 Department of Anesthesiology, Perioperative and Pain Medicine, School of Medicine, Stanford University, Stanford, California, USA.; 3 Laboratory of Pain and Signaling, Butantan Institute, Sāo Paulo, Brazil.; 4 Department of Anesthesiology, Intensive Care and Pain Medicine, University Hospital Muenster, Muenster, Germany.; 5 Department of Pathology,; 6 Stanford Diabetes Research Center, and; 7 Stanford Cardiovascular Institute, School of Medicine, Stanford University, Stanford, California, USA.; 8 Department of Obstetrics and Gynecology, School of Medicine, Emory University, Atlanta, Georgia, USA.

**Keywords:** Neuroscience, Vascular Biology, Anesthesiology, Mouse models, Pain

## Abstract

Pain signals are relayed to the brain via a nociceptive system, and in rare cases, this nociceptive system contains genetic variants that can limit the pain response. Here, we questioned whether a human transient receptor potential vanilloid 1 (TRPV1) missense variant causes a resistance to noxious stimuli and, further, whether we could target this region with a cell-permeable peptide as a pain therapeutic. Initially using a computational approach, we identified a human K710N TRPV1 missense variant in an otherwise highly conserved region of mammalian TRPV1. After generating a TRPV1^K710N^-knockin mouse using CRISPR/Cas9, we discovered that the K710N variant reduced capsaicin-induced calcium influx in dorsal root ganglion neurons. The TRPV1^K710N^ rodents also had less acute behavioral responses to noxious chemical stimuli and less hypersensitivity to nerve injury, while their response to noxious heat remained intact. Furthermore, blocking this K710 region in WT rodents using a cell-penetrating peptide limited acute behavioral responses to noxious stimuli and returned pain hypersensitivity induced by nerve injury to baseline levels. These findings identify K710 TRPV1 as a discrete site that is crucial for the control of nociception and provide insights into how to leverage rare genetic variants in humans to uncover fresh strategies for developing pain therapeutics.

## Graphical abstract

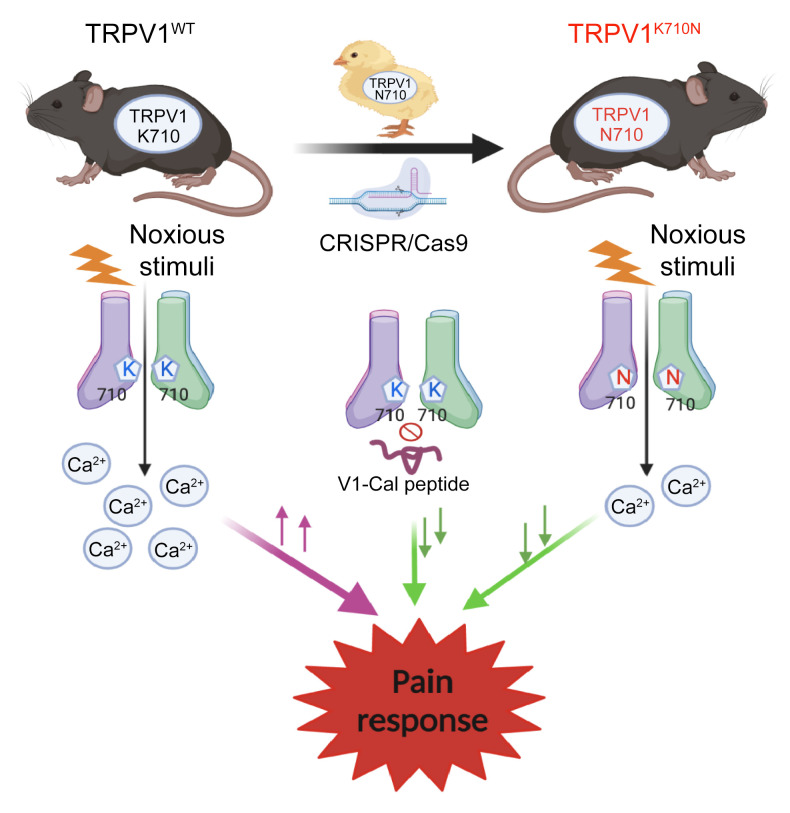

## Introduction

Pain afflicts over 1.5 billion individuals worldwide and costs more than $600 billion annually in United States alone (1). Secondary health problems from treating pain, including opioid abuse, dependence, and overdose, are fueling an opioid epidemic that is complicated by the present COVID-19 endemic (2). To address the human health problem of pain and the secondary impact caused by opioid dependence, addiction, and overdose, it is necessary to further understand how the nociceptive system perceives pain and how genetic variants within the nociceptive system may alter the intensity of this nociceptive signal. This would provide important insights that could assist with the development of therapeutics to limit pain.

In humans, documented missense or nonsense variants (as in the case for extremely rare Nav1.7 channel variants) can alter the pain response substantially. This modification of the nociceptive system and nociceptive response can result in responses at either end of the pain spectrum, whereby a gain-of-function variant can cause an extreme pain disorder and a loss-of-function variant a complete inability to experience pain (3–5). This example also highlights the notion that introducing a complete loss-of-function variant into a nociceptor could be detrimental, as sensing a noxious insult and withdrawing (such as when a hand is placed on a hot stove) is an important physiological function to limit cellular injury. Largely unexplored are the more than 1,000 reported human genetic variants within the transient receptor potential vanilloid 1 (TRPV1) channel, which is highly expressed in nociceptive sensory neurons that detect noxious stimuli, leading to pain perception and transmission (6). Past studies have shown that blocking the TRPV1 channel could alleviate pain behaviors, but most TRPV1 antagonists caused side effects such as abnormal temperature regulation and loss of a noxious heat response (7). In vitro site mutagenesis studies showed that point mutations can selectively eliminate TRPV1 activation but leave heat sensing intact, implying such modality-selective antagonists could be a more attractive approach for pain therapeutics (8, 9).

Recently, advances including a cryogenic electron microscopy (cryoEM) TRPV1 structure (10) and CRISPR/Cas9 technology for gene editing have provided the opportunity to further investigate human TRPV1 genetic variants. A CRISPR/Cas9 gene-editing system has recently been demonstrated to provide long-lasting pain relief via precisely targeted in vivo epigenetic repression of the Nav1.7 channel (11). This implies that CRISPR-based gene therapy could lead to an opioid-free way of treating pain (12). Additionally, one strategy to uncover human TRPV1 variants that may reduce pain responses could involve turning to birds. This is because within nature, avian species, unlike mammals, possess a TRPV1 receptor that is naturally resistant to noxious insults yet can still perceive pain and trigger protection from injuries such as a heart attack (13, 14). This is important to consider when investigating TRPV1 genetic variants, as recent evidence also suggests that TRPV1 in non-neuronal cells serves as an intracellular molecular sensor that protects against glucose-induced cellular stress or tissue ischemia (15, 16). Here, we hypothesize that by using the human TRPV1 missense variants identified through genetic sequencing, coupled with the genetic divergence in avian species relative to mammals, we can pinpoint a region for genetic manipulation of the mammalian TRPV1 receptor using CRISPR/Cas9 to reduce, yet not eliminate, TRPV1-mediated responses to pain without exacerbating cellular injury. If successful, we could then develop a cell-permeable peptide targeting this region to reduce acute and chronic pain responses.

## Results

### Identification of human missense TRPV1 variants that cause a genetic divergence to avian TRPV1.

**Figure 1:**
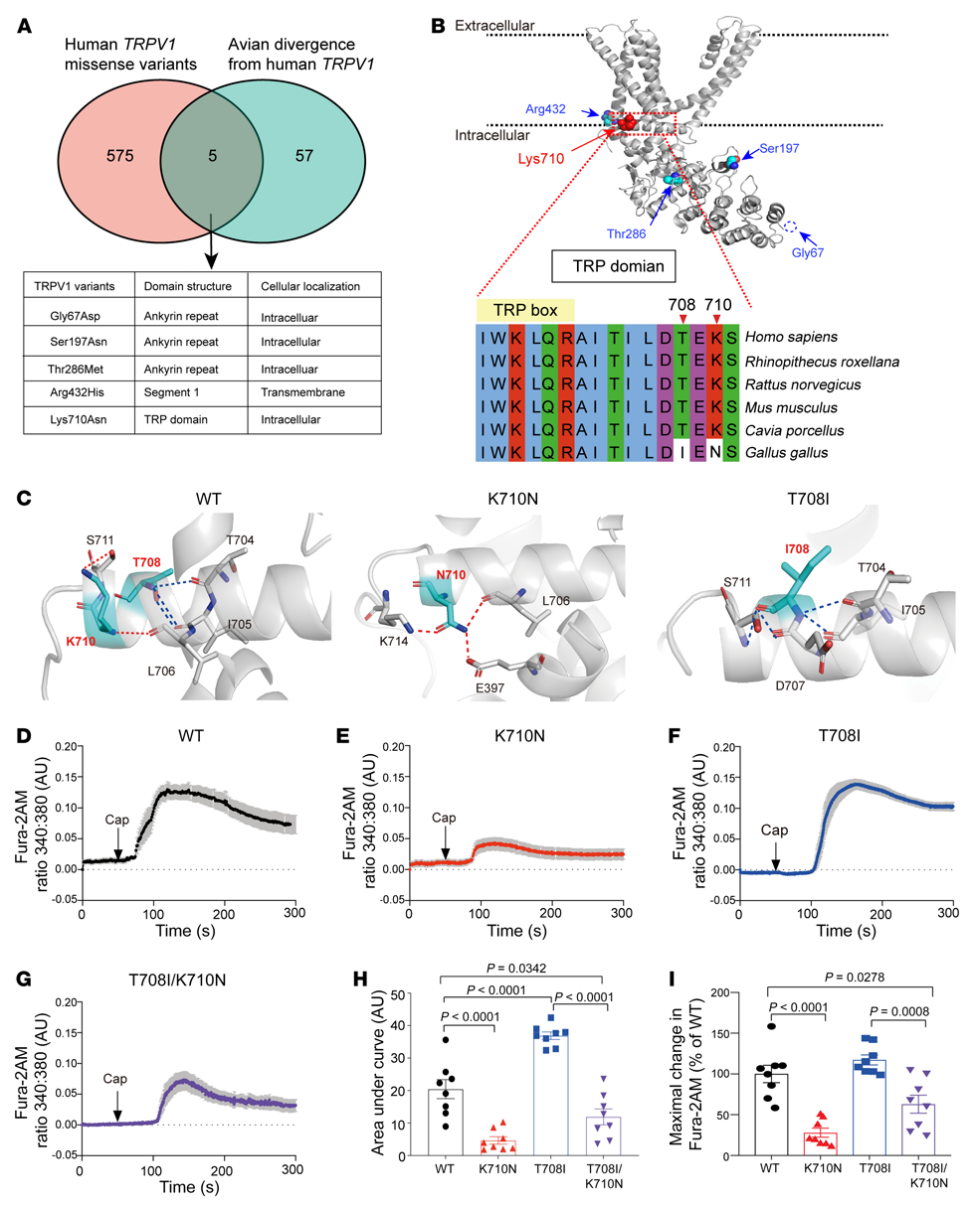
Identification of a human missense TRPV1 variant that reduces TRPV1-mediated calcium influx. (**A**) Venn diagram identifying 5 missense TRPV1 variants that intersect when examining the human TRPV1 missense variants from the gnomAD and avian genetic divergence from the human TRPV1 sequence. (**B**) Location of these 5 missense variants within the rat TRPV1 structure (PDB ID: 3J5P). Alignment of the mammalian and avian TRP domain (I696–S711 within the red dotted box; the unconserved amino acids 708 and 710 are shown in white). (**C**) 3D structure of WT TRPV1, K710N, and T708I based on the closed-state rat TRPV1 molecular model (PDB ID: 3J5P). Polar contacts are indicated in red or by blue dashed lines. (**D**–**G**) Calcium influx in response to 1 μM capsaicin (Cap) with (**D**) WT TRPV1, (**E**) K710N, (**F**) T708I, or (**G**) K710N/T708I TRPV1 mutations presented as a Fura-2AM ratio of 340:380 nm. (**H**) The AUC (total amount of calcium influx) and (**I**) percentage of maximal change in the Fura-2AM ratio for TRPV1 mutants relative to WT TRPV1 were calculated. *n* = 8 cells/group from 3 independent experiments. Data are expressed as the mean ± SEM. Significance was determined by 1-way ANOVA followed by Tukey’s post hoc test.

Within the Genome Aggregation Database (gnomAD), we identified 1,461 variants from 281,434 *TRPV1* alleles. We excluded the 259 synonymous variants and 542 other types of variants that did not result in a missense or loss of function. The remaining 580 missense variants and 80 predicted loss-of-function variants of human *TRPV1* were then compared with avian *TRPV1*, which genetically diverged much earlier from mammalian *TRPV1* (Supplemental Figure 1; supplemental material available online with this article; https://doi.org/10.1172/JCI163735DS1). To understand the basis for this divergence, multiple sequence alignment identified 62 amino acids that differed when comparing avian *TRPV1* with mammalian *TRPV1* (Supplemental Figure 2). When comparing this with the human *TRPV1* variants from the gnomAD, we identified 5 missense variants that caused human *TRPV1* to genetically revert back to avian *TRPV1* (Figure 1A).

For these 5 missense variants, we identified their location using the cryo-EM TRPV1 structure (Protein Databank [PDB] ID: 3J5P) (10). Of most interest was the variant identified within the C-terminus TRP domain (residues 687–711), which is located just after the sixth transmembrane segment and has been proposed to engage in channel gating (10). This region forms an intracellular α-helix parallel to the cell membrane (Figure 1B). Interestingly, just past the evolutionarily conserved TRP box (residues 696–701), avian TRPV1 diverges from that of mammalian species and has 2 residues, I708 and N710, that are different from the mammalian T708 and K710 residues (Figure 1B). When the structure is analyzed on the basis of cryo-EM TRPV1, the K710 residue forms hydrogen bonds with S711 and L706 (red dashed lines), whereas the T708 residue forms hydrogen bonds with T704 and I705 (blue dashed lines, Figure 1C).

To examine this further and to mimic the avian TRP domain sequence, we performed K710N and T708I substitutions using Chimera. The replacement of K710 with N710 causes changes in hydrogen bond interactions with neighboring amino acids. The N710 residue does not interact with S711, but instead contacts C-terminal K714 and E397 in the linker region (Figure 1C). In contrast, T708I substitution does not change its interactions with T704 and I705, while forming 2 more hydrogen bonds with S711 and D707 within the TRP domain (Figure 1C). These evolutionary and in silico data suggest that in avian species, the N710 and I708 residues, which are genetically divergent relative to mammalian K710 and T708, cause different structural changes in a region just past the TRPV1 TRP box that regulates TRPV1 gating (17). As such, we next determined whether these changes lead to functional differences in the TRPV1 channel.

### TRPV1 site-directed mutagenesis at K710 decreases capsaicin-induced calcium influx.

Since the K710N missense variant in humans is quite rare (1.6 × 10^–5^ frequency), we performed site-directed mutagenesis to explore whether K710N affects the channel function for TRPV1. To this end, we constructed K710N, T708I, and T708I/K710N mutations from a WT rat TRPV1 plasmid (Supplemental Table 1) and transfected them into H9C2 cells, which lack endogenous TRPV1 cell-surface expression. The K710N-transfected cells treated with capsaicin (1 μM, a selective and specific TRPV1 agonist) reduced the total amount of calcium influx (AUC) by 4.4-fold compared with WT TRPV1–transfected cells (Figure 1, D, E, and H; 4.66 ± 1.14 vs. 20.45 ± 2.95 AU, respectively, *P* < 0.0001). In contrast, the T708I mutation caused a 1.8-fold increase in intracellular calcium influx relative to WT TRPV1 cells (Figure 1, D, F, and H; 36.90 ± 1.15 vs. 20.45 ± 2.95 AU, respectively, *P* < 0.0001). However, the T708I/K710N double mutation caused 1.7-fold less calcium influx than did the WT TRPV1 cells following capsaicin stimulation (Figure 1, D, G, and H; 11.93 ± 2.44 vs. 20.45 ± 2.95 AU, respectively, *P* = 0.0342).

The peak intracellular calcium levels after capsaicin was also decreased in K710N TRPV1 cells relative to levels in WT TRPV1 cells (Figure 1I; 286% vs. 100% ± 11%, percentage of WT, respectively, *P* < 0.0001). The cells transfected with T708I TRPV1 had a peak intracellular calcium rise similar to that of WT TRPV1 cells, while the peak was lower for the T708I/K710N TRPV1 double mutation (Figure 1I; T708I: 117% ± 6%, T708I/K710N: 63% ±11% vs. 100% ± 11% in WT TRPV1, percentage of WT). Together, these data suggest that K710N TRPV1 reduced capsaicin-induced calcium influx in transiently transfected H9C2 cells.

### Generation of CRISPR/Cas9-edited TRPV1^K710N^-knockin mice.

**Figure 2:**
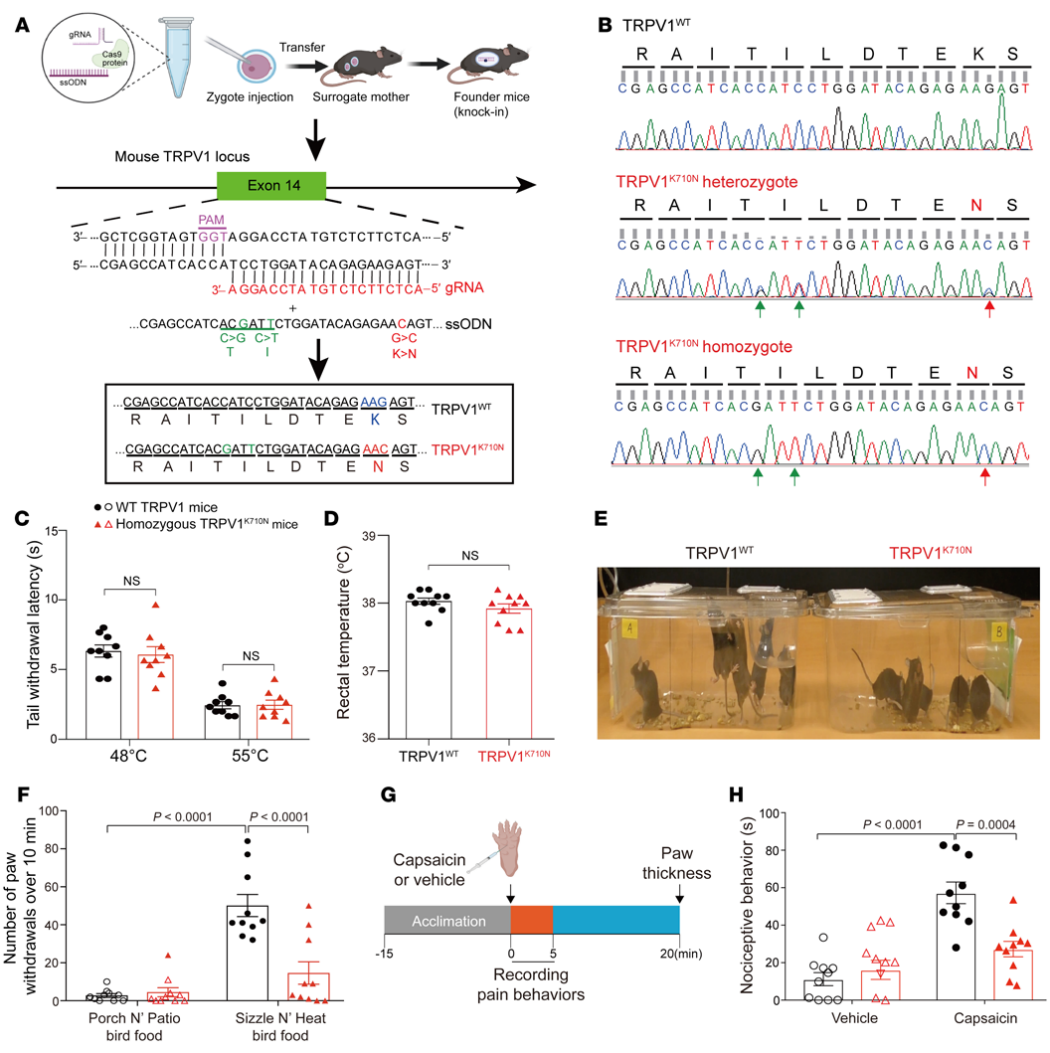
Construction of CRISPR/Cas9-edited TRPV1^K710N^-knockin mice followed by thermal and behavioral testing. (**A**) Schematic diagram for the generation of CRISPR/Cas-9 edited TRPV1^K710N^-knockin mice. In the design of gRNA, the ssODNs lead to the K710N mutation (red) and 2 silent mutations (green) in the PAM (purple). (**B**) Representative DNA-Seq for WT TRPV1 and TRPV1^K710N^ heterozygotes (double peak) or TRPV1^K710N^ homozygotes (single peak). Red arrows indicate the designed mutation; green arrows indicate the silent mutations. (**C**) Tail withdrawal latency (seconds) in response to hot water at 48°C and 55°C (*n* = 9/group). (**D**) Body temperature detected with a rectal temperature probe in WT TRPV1 and homozygous TRPV1^K710N^ mice (*n* = 10/group). (**E**) Representative image showing the difference in response to stepping on capsaicin-laced food for WT TRPV1 and TRPV1^K710N^ mice. (**F**) Paw withdrawal behavior of WT TRPV1 and TRPV1^K710N^ mice exposed to capsaicin-laced bird food (Sizzle N’ Heat Bird food) or regular bird food (Porch N’ Patio Bird food) (*n* = 10/group). (**G**) Experimental protocol for nociceptive behavior by intraplantar capsaicin injection into WT TRPV1 and TRPV1^K710N^ mice. (**H**) Nociceptive behavior (paw licking/flicking) (*n* = 10/group). Data are expressed as the mean ± SEM. Significance was determined by unpaired *t* test (**C** and **D**) and 2-way ANOVA followed by Tukey’s post hoc test (**F** and **H**).

To determine whether the K710N TRPV1 point mutation alters responses to noxious insults including capsaicin in vivo, we generated a TRPV1^K710N^-knockin mouse using CRISPR/Cas9 (Figure 2A). Three guide RNAs (gRNAs) were designed near the K710 site located in exon 14 of the mouse TRPV1 locus (Supplemental Table 2). Both gRNA1 and gRNA2 could cut efficiently, and gRNA1 was used for mouse zygote injection. The donor single-stranded oligodeoxynucleotide (ssOD) contains a missense mutation for K710N (red base) and 2 silent mutations (green bases) in the gRNA protospacer adjacent motif (PAM) site (purple bases) to block the PAM sequence and thereby prevent recutting of the corrected mutated alleles by gRNA-Cas9 (18) (Figure 2A). The gRNA, together with the mutation containing ssODNs and Cas9 protein, was coinjected into the pronucleus of C57BL/6J zygotes and implanted into recipient mothers. A total of 16 pups were born, and CRISPR/Cas9-mediated editing occurred in all pups as determined by a T7 endonuclease I (T7EI) cleavage assay (Supplemental Figure 3, A and B). Among 16 pups, 6 mice bearing the designed mutation were identified by DNA sequencing (Supplemental Figure 3C). Of those, we identified a founder mouse without a nonhomologous end-joining (NHEJ) mutation and with only the desired G>C point mutation to produce a TRPV1 K710N mutation. We backcrossed the TRPV1^K710N^ mouse with C57BL/6J WT mice and then inbred them for heterozygous (double peak) and homozygous (single peak) offspring, which were verified by DNA sequencing (Figure 2B). After intercrossing for 3 generations, we used the fourth generation TRPV1^K710N^ homozygous mice for testing. Eighth-generation offspring of TRPV1^K710N^ mice were sent to Charles River Laboratories (France) for rederivation, and vascular studies were performed in Germany using TRPV1^K710N^ homozygous mice. The TRPV1^K710N^ mice were also generated at Shanghai Model Organisms Center Inc. and used to reproduce a portion of the DRG studies in-house. The TRPV1^K710N^ mice were viable and fertile and exhibited normal motor activity when subjected to rotarod testing relative to WT mice (Supplemental Figure 4, A and B). In addition, the TRPV1^K710N^ mice showed intact responses to noxious heat (48°C and 55°C), with similar withdrawal latencies relative to WT mice in a tail immersion test (Figure 2C; 48°C: 7.7 ± 0.9 vs. 6.7 ± 0.9 s, *P* = 0.4561). The body temperature detected by a rectal thermal probe also did not differ between the WT TRPV1 and TRPV1^K710N^ mice (Figure 2D; 38.0°C ± 0.03°C vs. 37.9°C ± 0.07°C, *P* = 0.2006). When considering sex as a biological variable, no differences were seen in these temperature studies (Supplemental Figure 5, A and B).

### TRPV1^K710N^-knockin mice are resistant to capsaicin-elicited nocifensive behavior.

Next, we challenged the WT TRPV1 and TRPV1^K710N^ mice with bird food laced with capsaicin. This type of food is typically used by chicken farmers, as the capsaicin prevents mammals, such as squirrels, from eating the food, while the chickens eat ad libitum. Unexpectedly, by placing the bird food on the floor of the cage, this resulted in a rodent behavioral response, including rodents lifting their paws off the bird food in response to the dermal absorption of capsaicin. We quantified this difference in behavioral responses between the WT TRPV1 and TRPV1^K710N^ mice by monitoring paw withdrawal (in which at least 3 paws were on the cage wall or jumping) after exposure to the bird food with capsaicin. When exposed to the bird food containing capsaicin (Sizzle N’ Heat), the WT mice exhibited more nocifensive behavior compared with the TRPV1^K710N^ mice (Figure 2E and Supplemental Video 1). Paw withdrawal from the food was markedly increased for the WT TRPV1 mice relative to the TRPV1^K710N^ mice (Figure 2F; 50 ± 6 vs. 15 ± 6 instances of paw withdrawal/10 min, respectively, *P* < 0.0001). In contrast, when exposed to the bird food without capsaicin (Porch N’ Patio), none of the mice in either group exhibited this behavior (Supplemental Video 2). The paw withdrawal behavior was similar between WT TRPV1 and TRPV1^K710N^ mice (Figure 2F). When considering sex as a variable, no differences in behavior were observed between the male and female mice (Supplemental Figure 5C).

We then examined the nociceptive behavior triggered by intraplantar capsaicin injection (Figure 2G). Nociceptive behaviors (paw-licking/flinching) after capsaicin injection were reduced in TRPV1^K710N^ mice relative to WT TRPV1 mice (Figure 2H; 27 ± 4 vs. 57 ± 6 s, respectively, *P* = 0.0004). The WT TRPV1 mice exhibited nociceptive behaviors including paw-licking and flinching over 2-minutes after capsaicin injection (Supplemental Video 3). In comparison, TRPV1^K710N^ mice showed less nociceptive behaviors, with most occurring in the first 30 seconds of capsaicin injection (Supplemental Video 4). After vehicle injection, we observed no significant difference in paw-licking/flinching times between the 2 groups (Figure 2H). When considering sex as a biological variable, no differences were noted between the male and female rodents (Supplemental Figure 5D). We also evaluated the pain behavior induced by Brp-LPA, an analog of lysophosphatidic acid (LPA) that induces acute pain by directly interacting with TRPV1 at the K710 site (19). Nociceptive behaviors induced by Brp-LPA were markedly decreased in TRPV1^K710N^ mice compared with the behaviors of WT TRPV1 mice (Supplemental Figure 6). As we did not identify any sex differences between the male and female mice, we used male mice for the remainder of the study.

**Figure 3:**
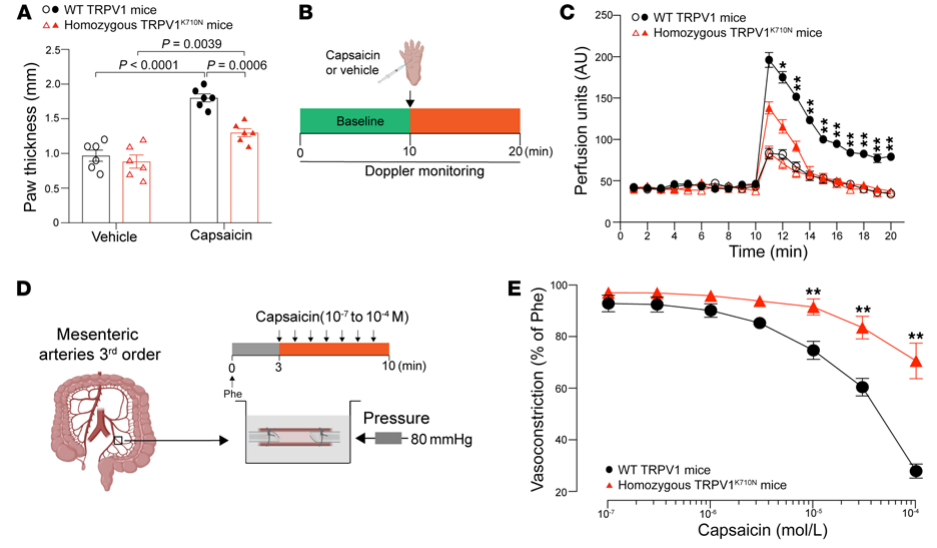
Vascular response in TRPV1^K710N^-knockin mice. (**A**) Paw thickness and (**B** and **C**) blood flow induced by injection of capsaicin or vehicle into the paws of WT TRPV1 and TRPV1^K710N^ mice (*n* = 6/group). (**D** and **E**) Pressure myography showing the vascular response to capsaicin in WT TRPV1 (*n* = 7) and TRPV1^K710N^ mice (*n* = 8). Data are expressed as the mean ± SEM. *P < 0.05 and **P < 0.01, for capsaicin-treated WT TRPV1 mice versus capsaicin-treated TRPV1^K710N^ mice, by 2-way RM ANOVA followed by Bonferroni’s post hoc test (**C** and **E**).

Capsaicin injection induces neurogenic inflammation, causing vasodilation and tissue edema (20). Following intraplantar capsaicin injection, we found that paw thickness was also reduced in TRPV1^K710N^ mice relative to that of WT TRPV1 mice (Figure 3A). Blood flow, as measured by laser Doppler flowmetry, was also markedly increased following capsaicin injection into WT TRPV1 mice and was less in TRPV1^K710N^ mice (Figure 3, B and C). Pressure myography of mesenteric resistance arteries from WT TRPV1 and TRPV1^K710N^ mice also revealed that arteries from TRPV1^K710N^ mice had a reduced vasodilatory response to capsaicin compared with those of WT TRPV1 mice (Figure 3, D and E).

**Figure 4:**
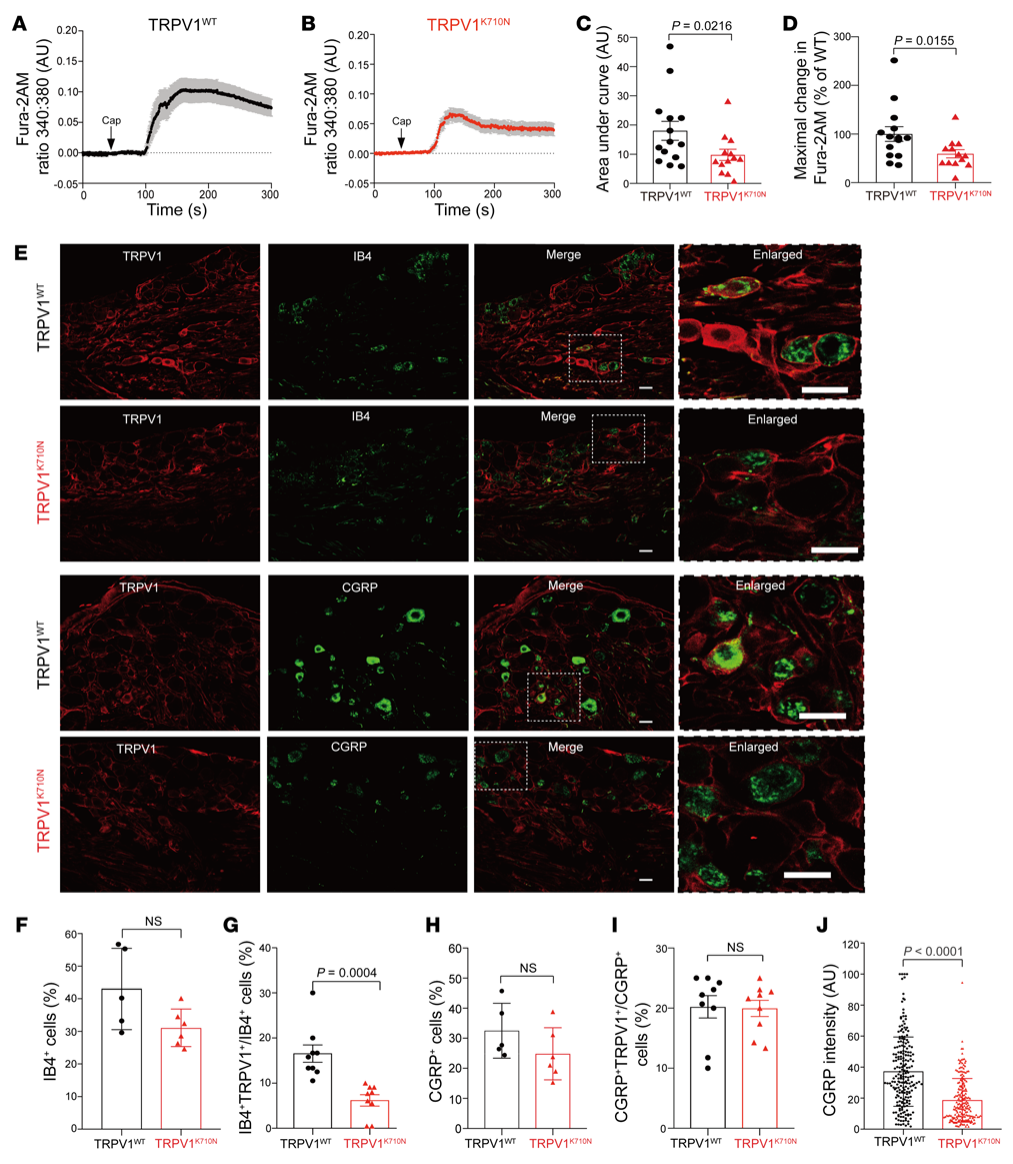
Characterization of DRG neurons from TRPV1^K710N^-knockin mice. (**A** and **B**) Fura-2AM in response to capsaicin treatment in DRG neurons from WT TRPV1 and TRPV1^K710N^ mice, shown as changes in the Fura-2AM ratio. (**C**) AUC (total amount of calcium influx) and (**D**) percentage of maximal change in calcium influx for TRPV1^K710N^ mice (*n* = 13) relative to WT TRPV1 mice (*n* = 14). Data are from 3 independent experiments. (**E**) Representative images showing colocalization of TRPV1 with IB4 and CGRP. Scale bars: 20 μm. (**F**–**I**) Total percentage of (**F**) IB4^+^ cells and percentage of (**G**) IB4^+^ neurons expressing TRPV1. Total percentage of (**H**) CGRP^+^ cells and percentage of (**I**) CGRP^+^ neurons expressing TRPV1. *n* = 5–9 from 3 independent experiments. (**J**) Fluorescence intensity of CGRP in TRPV1^K710N^ neurons (*n* = 202) versus WT TRPV1 neurons (*n* = 208) from 3 mice in each group. Data are expressed as the mean ± SEM and were analyzed using a 1-tailed (**C** and **D**) or 2-tailed (**F**–**J**) *t* test.

We then cultured primary DRG neurons from WT TRPV1 and TRPV1^K710N^ mice and observed different responses to capsaicin-induced calcium influx in these mice (Figure 4, A and B). The capsaicin-induced AUC was reduced in TRPV1^K710N^ DRGs compared with the AUC for WT TRPV1 DRGs (Figure 4C; 9.8 ± 1.9 vs. 18.0 ± 3.3 AU, respectively, *P* = 0.022). The peak intracellular change in Fura-2 acetoxymethyl (Fura-2AM) was also 41% lower in TRPV1^K710N^ DRG cells relative to levels in WT TRPV1 DRG cells (Figure 4D; 59% ± 8% vs. 100% ± 15%, percentage of WT, respectively, *P* = 0.016). These findings in TRPV1^K710N^ and WT TRPV1 mice were also reproduced at a separate laboratory (Supplemental Figure 7, A–D).

We next examined the colocalization of TRPV1 with isolectin B4 (IB4) and calcitonin gene–related peptide (CGRP) in DRG neurons from WT TRPV1 and TRPV1^K710N^ mice (Figure 4E). The total percentage of IB4^+^ neurons was not different between WT TRPV1 and TRPV1^K710N^ mice (Figure 4F). However, the number of IB4^+^ neurons expressing TRPV1 in TRPV1^K710N^ mice was significantly reduced compared with that detected in WT TRPV1 mice (Figure 4G; 6.2% ± 1.3% vs. 16.5% ± 1.9%, respectively, *P* = 0.0004). Although there were no differences in the total number of CGRP neurons (Figure 4H) and the number of CGRP^+^ neurons expressing TRPV1 (Figure 4I), CGRP intensity was decreased in DRG neurons from TRPV1^K710N^ mice relative to that of DRG neurons from WT TRPV1 mice (Figure 4J).

### TRPV1^K710N^ mice have attenuated hypersensitivity from nerve injury.

**Figure 5:**
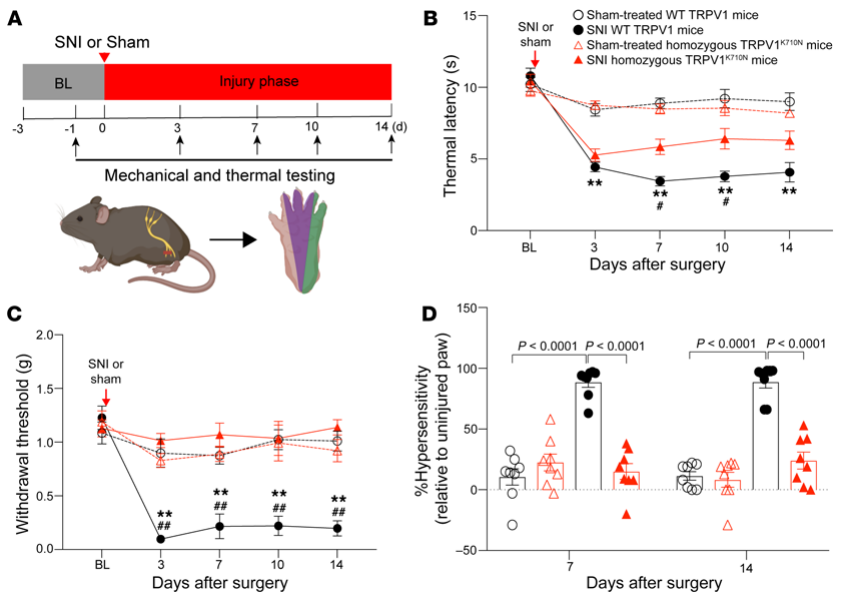
TRPV1^K710N^-knockin mice are resistant to nerve injury behavior. (**A**) Experimental protocol for the SNI model. Purple indicates the tibial innervated area; green indicates the sural territory (test area). (**B**) Thermal latency results for the sham and SNI groups. (**C**) Withdrawal thresholds for mechanical stimuli for the sham and SNI groups. (**D**) Percentage of hypersensitivity for mice in the sham and SNI groups. Data are expressed as the mean ± SEM (*n* = 8/group). **P < 0.01, WT TRPV1 SNI versus WT TRPV1 sham; ^#^*P* < 0.05 and ^##^*P* < 0.01, WT TRPV1 SNI versus TRPV1^K710N^ SNI. Two-way repeated-measures (RM) ANOVA followed by Bonferroni’s post hoc test. BL, baseline.

WT TRPV1 and TRPV1^K710N^ mice were subjected to spared nerve injury (SNI) (21), and mechanical and thermal sensitivity were measured at designated time points (Figure 5A). Nerve injury shortened the thermal latency in both groups of mice, but thermal hypersensitivity in TRPV1^K710N^ mice was partly recovered beginning on the day 7 after surgery. We observed no significant difference between the sham-treated and SNI group of TRPV1^K710N^ mice on days 10 and 14 after surgery (Figure 5B). WT TRPV1 mice had a significantly lower mechanical withdrawal threshold on day 3 after SNI relative to rodents that underwent the sham procedure (Figure 5C; 0.10 ± 0.03 vs. 0.90 ± 0.13 g, *P* = 0.0016), with continued mechanical hypersensitivity throughout the 2 weeks of testing. In contrast, TRPV1^K710N^ mice showed less of an effect than the WT TRPV1 mice when assessed for the withdrawal threshold over the course of 2 weeks (Figure 5C; 1.14 ± 0.07 vs. 0.20 ± 0.07 g on day 14, respectively, *P* < 0.0001). Furthermore, the percentage of mechanical hypersensitivity relative to the uninjured paw was significantly reduced in TRPV1^K710N^ mice relative to that of WT TRPV1 mice (Figure 5D; 24% ± 7% vs. 89% ± 5 % on day 14, respectively, *P* < 0.0001). These data suggest that the K710N mutation reduced nerve injury–induced pain hypersensitivity.

### TRPV1^K710N^-knockin mice are resistant to cellular injury.

**Figure 6:**
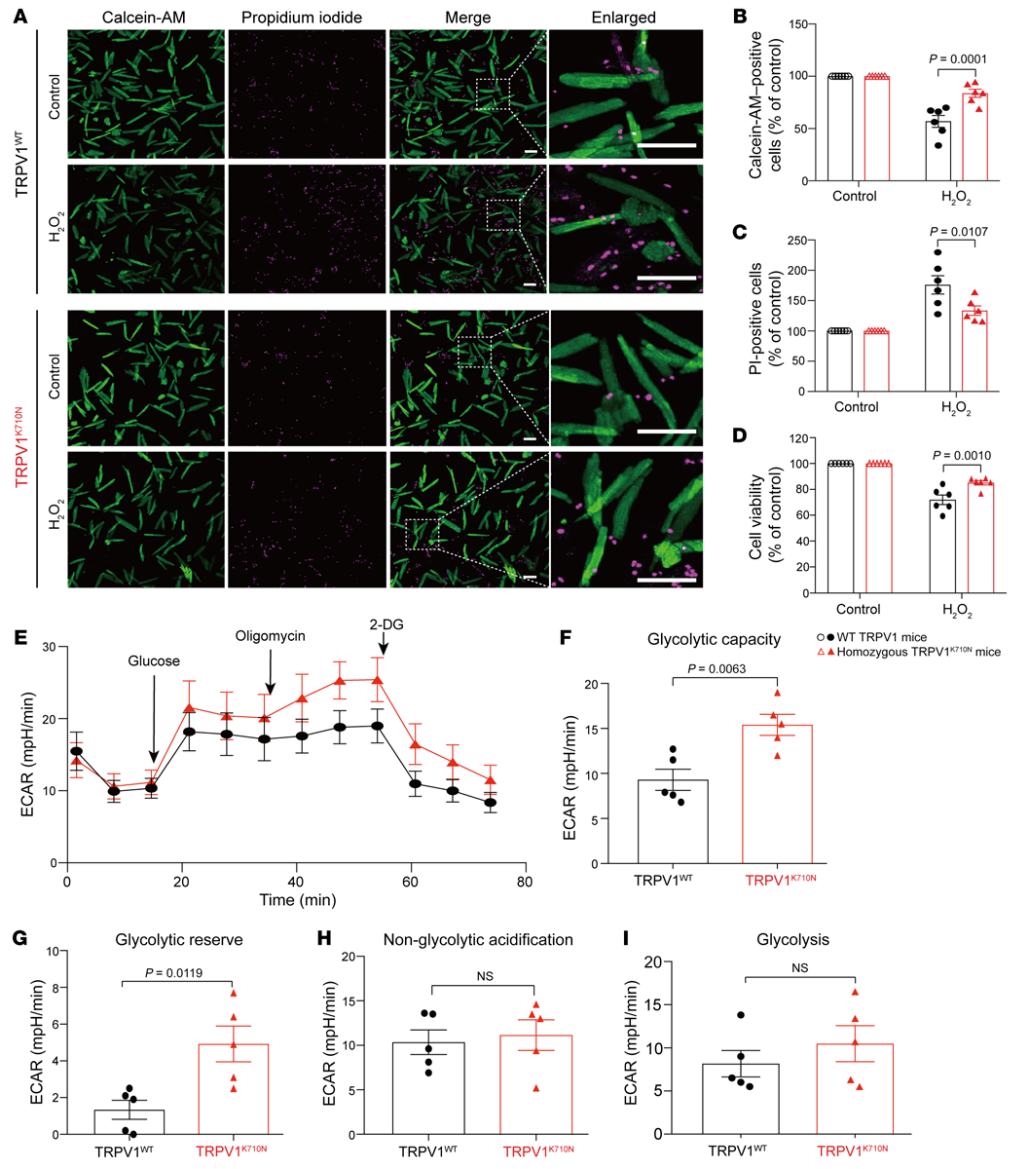
TRPV1^K710N^-knockin mice have less cellular injury and improved glycolytic function. (**A**) Representative images of calcein AM–stained (green) and PI-stained (magenta) cardiomyocytes with or without H_2_O_2_ treatment. Scale bars: 100 μm. (**B**) Calcein AM–stained viable cells and (**C**) PI-stained dead cells were quantified by the average fluorescence intensity. (**D**) Cell viability by MTT assay. The value of the control cells in WT TRPV1 or TRPV1^K710N^ cells was set at 100% (*n* = 6/group). (**E**–**I**) Glycolysis stress tests in WT TRPV1 and TRPV1^K710N^ cardiomyocytes: (**E**) ECAR, (**F**) glycolytic capacity, (**G**) glycolytic reserve, (**H**) nonglycolytic acidification, and (**I**) glycolysis (*n* = 5/group). Data are expressed as the mean ± SEM. Significance was determined by 2-way ANOVA followed by Tukey’s post hoc test (**B**–**D**) and unpaired, 2-tailed *t* test (**F**–**I**).

Besides transmitting a nociceptive signal to the brain, the TRPV1 channel also plays an important role in regulating cellular injury that is independent of the nervous system (22–24). We further examined whether the TRPV1 K710N variant, as it limits calcium-induced cellular influx, exacerbates cellular injury in isolated cardiomyocytes (that are devoid of a nervous system) when subjected to hydrogen peroxide (H_2_O_2_) or hypoxia/reoxygenation. After exposure to H_2_O_2_, the percentage of calcein-AM–stained viable cells was higher in TRPV1^K710N^ cells relative to that in WT TRPV1 cells (Figure 6, A and B; 84% ± 4% vs. 57% ± 6%, % of control, *P* = 0.0001). In contrast, the percentage of propidium iodide–stained (PI-stained) dead cells were reduced in TRPV1^K710N^ cells compared with the percentage in WT cells (Figure 6, A and C; 133% ± 8% vs. 176% ± 15%, percentage of control, *P* = 0.0107). Additionally, an MTT assay showed that the viability of TRPV1^K710N^ cells was higher than that of WT cells (Figure 6D; 85% ± 2% vs. 72% ± 4 %, percentage of control, respectively, *P* = 0.0010). Following hypoxia/reoxygenation injury, TRPV1^K710N^ cardiomyocytes had more calcein-AM–stained viable cells and fewer PI-stained dead cells, as well as increased cell viability relative to WT cells (Supplemental Figure 8, A–D).

We further examined the glycolytic function of the cardiomyocytes using a Seahorse extracellular flux analyzer as a mechanism to determine why TRPV1^K710N^ cells are tolerant to cellular injury. The response to glycolytic stress, as evaluated by measuring the ECAR under glucose, oligomycin, and 2-DG, was different between WT and TRPV1^K710N^ cells (Figure 6E). When analyzing glycolytic function, we found that the glycolytic capacity was significantly increased in TRPV1^K710N^ cardiomyocytes compared with WT cardiomyocytes (Figure 6F; ECAR: 15.40 ± 1.18 vs. 9.30 ± 1.17 mPH/min, *P* = 0.0063). The glycolytic reserve of TRPV1^K710N^ cardiomyocytes was also higher than that of WT cells (Figure 6G; ECAR: 4.92 ± 0.98 vs. 1.34 ± 0.52 mPH/min, *P* = 0.0119). No differences between the 2 mouse groups were observed for nonglycolytic acidification and glycolysis (Figure 6, H and I).

Given that the TRPV1^K710N^ mice had an elevated glycolytic reserve and that the primary fuel source for the brain is glucose, we also questioned whether these mice were resistant to stroke in a focal cerebral ischemia model of 1-hour transient middle cerebral artery occlusion (MCAO) followed by 24 hours of reperfusion. The cerebral infarct size in TRPV1^K710N^ mice was significantly reduced relative to that seen in WT mice (Supplemental Figure 8, E and F; 18% ± 3% vs. 29% ± 3 %, respectively, *P* = 0.0234). Together, these observations demonstrate that TRPV1^K710N^ mice had the beneficial effect of enhanced glycolytic function and decreased injury from oxidative stress and ischemia, even though there was a reduced behavioral response to pain.

### A peptide targeting the TRPV1 K710 region limits capsaicin-induced nocifensive behavior and chronic pain from nerve injury.

Since TRPV1^K710N^ mice did not show exacerbated organ injury despite reduced, but not eliminated, nocifensive responses to noxious insults, we used the native mammalian TRPV1 structure as a model to develop a peptide (_701_RAITILDTEKS_711_) spanning the α-helix that contained K710, which is just past the evolutionarily conserved TRP box. As this region is an intracellular helix that runs parallel to the membrane, the peptide was conjugated to TAT_47–57_ for intracellular entry, and the peptide was named V1-cal (23).

**Figure 7:**
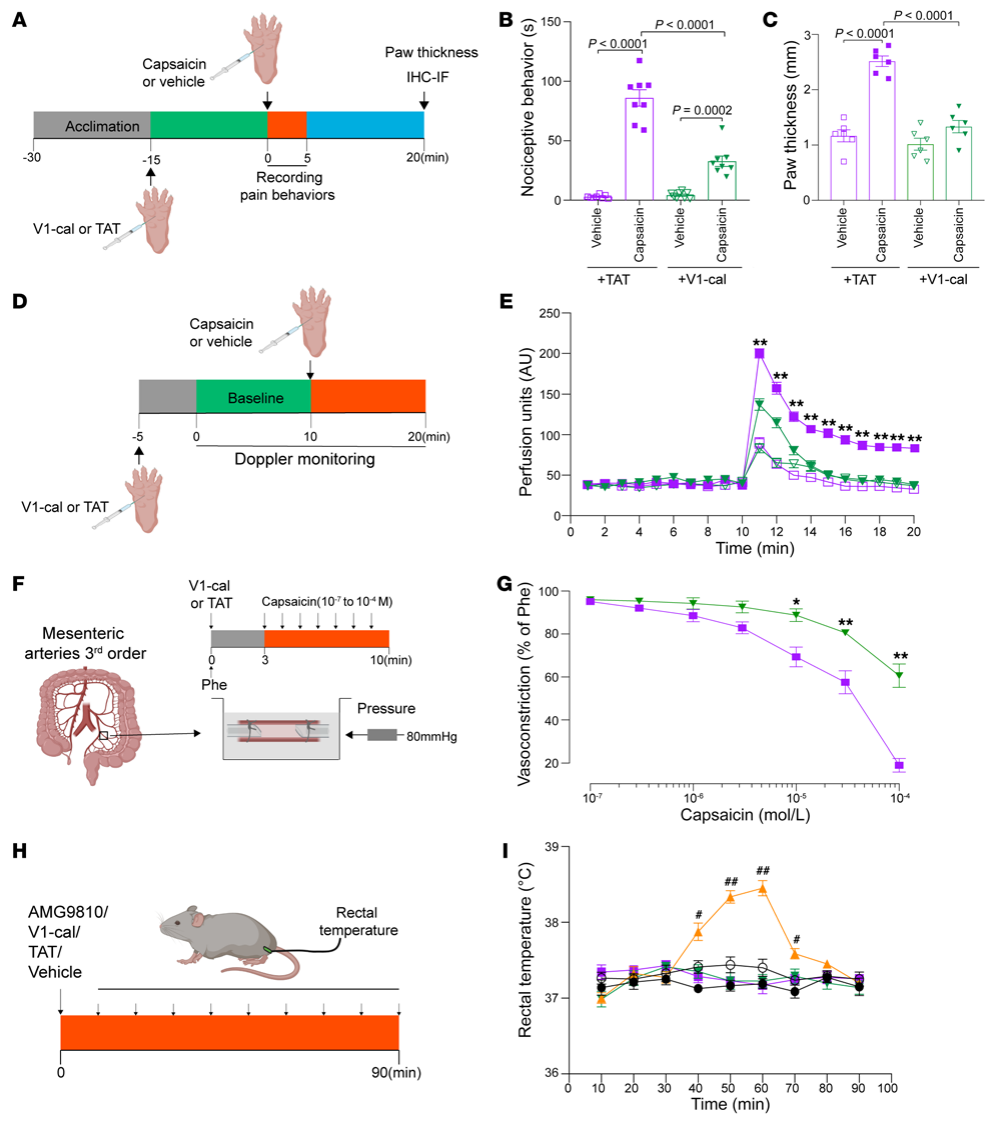
A peptide targeting K710 acutely prevents capsaicin-induced nocifensive behavior and vasodilation without thermal changes. (**A**) Experimental protocol for peptide treatment before intraplantar capsaicin injection in WT TRPV1 mice. IF, immunofluorescence. (**B**) Nociceptive behavior and (**C**) paw thickness induced by injection into the paw of capsaicin after V1-cal or TAT_47–57_ treatment. *n* = 8/group. (**D** and **E**) Paw blood flow induced by injection of capsaicin (solid symbols) or vehicle (hollow symbols) in the presence of V1-cal (green) or TAT_47–57_ (purple). (**F** and **G**) Pressure myography of mesenteric resistance arteries in response to capsaicin in the presence of V1-cal (green) or TAT_47–57_ (purple). *n* = 6–8/group. (**H** and **I**) Body temperature detected by a rectal temperature probe in WT TRPV1 mice followed by intraperitoneal injection of V1-cal (green), TAT_47–57_ (purple), AMG9810 (yellow), peptide vehicle (saline, solid black circles), or AMG9810 vehicle (5% ethanol in saline, hollow black circles). *n* = 7–8/group. Data are expressed as the mean ± SEM. *P < 0.05 and **P < 0.01, V1-cal plus capsaicin group versus TAT_47–57_ plus capsaicin group; ^#^*P* < 0.05 and ^##^*P* < 0.01, AMG9810 group versus the vehicle group. Significance was determined by 2-way ANOVA followed by Tukey’s post hoc test (**B** and **C**) and 2-way RM ANOVA analysis followed by Bonferroni’s post hoc test (**E**, **G**, and **I**).

V1-cal or TAT_47–57_ was injected into the paw 15 minutes before capsaicin or vehicle intraplantar injection (Figure 7A). Treatment with either TAT_47–57_ or V1-cal alone did not elicit a behavioral response in WT TRPV1 rodents (Figure 7B; 2.9 ± 0.5 and 4.3 ± 0.9 s, respectively). When WT TRPV1 mice were subjected to acute capsaicin treatment, V1-cal substantially reduced the nociceptive response to capsaicin relative to that seen in the TAT_47–57_ vehicle–treated rodents (Figure 7B; 32.8 ± 4.4 vs. 86.0 ± 6.9 s, respectively, *P* < 0.001). Brp-LPA–induced responses in WT TRPV1 mice were also reduced by pretreatment with V1-cal (Supplemental Figure 9). Moreover, V1-cal reduced capsaicin-induced paw swelling compared with TAT_47–57_ (Figure 7C). V1-cal also reduced capsaicin-mediated blood-flow changes relative to TAT_47–57_ treatment (Figure 7, D and E). The vasodilative response to capsaicin was also markedly attenuated by V1-cal but not TAT_47–57_ (Figure 7, F and G). Since TRPV1 antagonists can trigger an increase in body temperature, we further measured the rectal temperature after V1-cal or TAT_47–57_. Neither V1-cal nor TAT_47–57_ had much effect on body temperature compared with AMG9810, a TRPV1 antagonist, which increased the temperature after injection (Figure 7, H and I).

**Figure 8:**
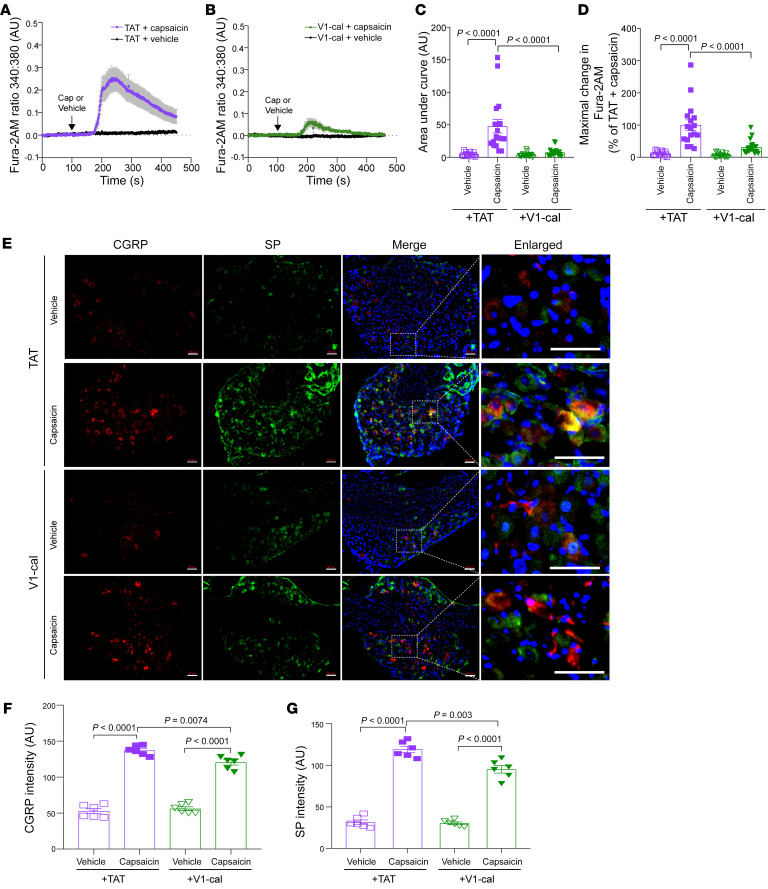
Effect of V1-cal or TAT_47–57_ on DRG neurons. (**A** and **B**) WT DRG neurons were treated with TAT_47–57_ or V1-cal, with and without capsaicin. (**C**) AUC (total amount of calcium influx) and (**D**) percentage of maximal change in Fura-2AM following capsaicin-stimulated calcium influx relative to TAT_47–57_. *n* = 16–18 DRG cells from 3 independent experiments. (**E**) Immunostaining for SP and CGRP in DRG tissues following capsaicin or vehicle injection in the presence of V1-cal or TAT_47–57_. Scale bars: 50 μm. Quantitative analysis of (**F**) CGRP intensity and (**G**) SP intensity in each group. *n* = 6/group. Significance was determined by 2-way ANOVA followed by Tukey’s post hoc test (**C**, **D**, **F**, and **G**). *n* = 6/group.

We then cultured primary DRG neurons from WT TRPV1 mice and perfused DRG neurons with V1-cal (1 μM) or TAT_47–57_ (1 μM) 10 minutes prior to capsaicin (1 μM, 15 s) treatment and up until the end of capsaicin treatment. We observed different responses to capsaicin-induced calcium influx in TAT_47–57_–treated relative to V1-cal–treated DRGs (Figure 8, A and B). The capsaicin-induced AUC was reduced in V1-cal–treated DRGs compared with TAT_47–57_–treated DRGs (Figure 8C; 7.6 ± 1.5 vs. 47.8 ± 10.3 AU, respectively, *P* < 0.0001). The peak intracellular change in Fura-2AM was also 69% lower in V1-cal–treated DRG cells than in TAT_47–57_–treated DRG cells (Figure 8D; 31% ± 5% vs. 100% ± 16%, percentage of TAT_47–57_ plus capsaicin, respectively, *P* = 0.0001).

As capsaicin can alter the expression of neuropeptides in TRPV1^+^ sensory neurons (25), we examined the expression of substance P (SP) and CGRP in DRG tissues 20 minutes after intraplantar capsaicin injection into TAT_47–57_–treated and V1-cal–treated mice. We found that the capsaicin-induced upregulation of SP and CGRP in DRG neurons was decreased in the V1-cal–treated group when compared with the TAT_47–57_ –treated group (Figure 8, E–G).

As drugs targeting TRPV1 may lead to unwanted temperature changes, we implanted mice with a subcutaneous temperature probe to measure body temperature in awake, freely moving mice. After acquiring 2 days of baseline temperature data, we implanted rodents with osmotic pumps administering TAT_47–57_ vehicle or V1-cal. Body temperature measured over the subsequent 12 days showed that V1-cal had no apparent effect on rodent body temperature (Supplemental Figure 10).

**Figure 9:**
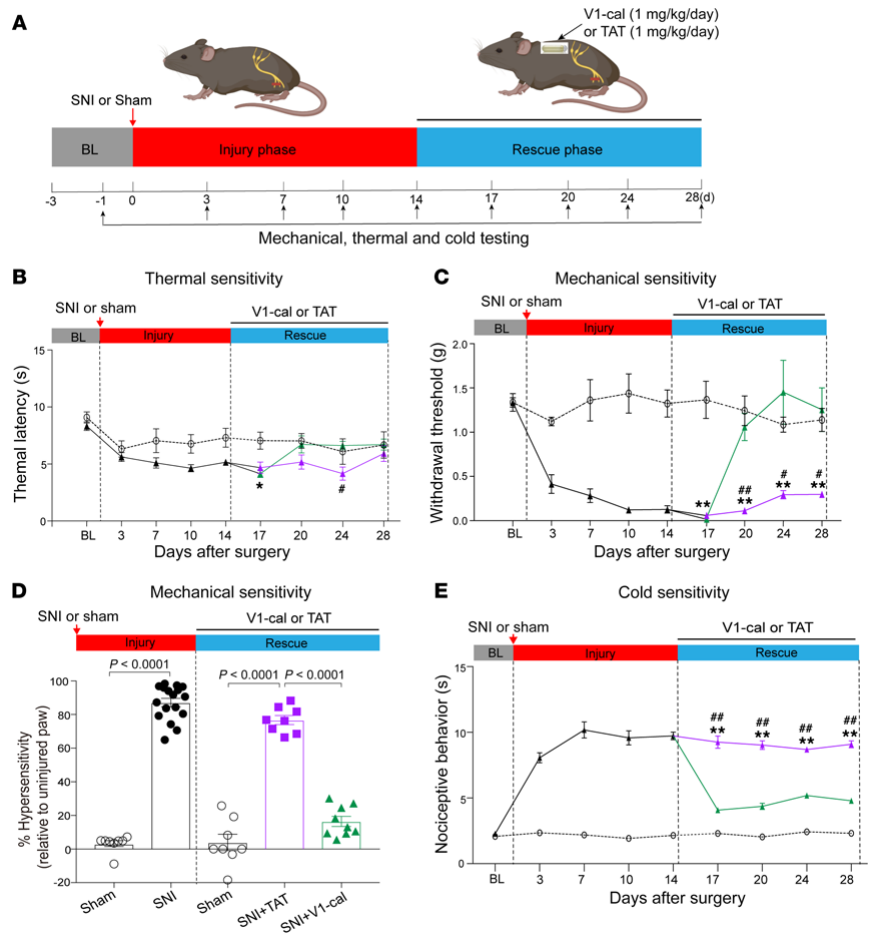
V1-cal rescues nocifensive behavior in WT TRPV1 mice after SNI. (**A**) Experimental protocol for SNI model with peptide treatment. Rodents underwent SNI and were assessed for 2 weeks (injury phase). After the injury phase, osmotic pumps were implanted to deliver V1-cal or TAT_47–57_ for 2 weeks (rescue phase). (**B**) Thermal latency for sham-treated (dashed line) and SNI (solid line) mice. SNI mice were treated with V1-cal (green) or TAT_47–57_ (purple) during the rescue phase. (**C**) Threshold of withdrawal from mechanical stimuli for sham-treated (dashed line) and for SNI (solid line) mice. SNI mice were treated with V1-cal (green) or TAT_47–57_ (purple) during the rescue phase. (**D**) Percentage of hypersensitivity relative to the uninjured paw for sham-treated (*n* = 8) and SNI (*n* = 17) mice treated with V1-cal (*n* = 9) or TAT_47–57_ (*n* = 8) during the injury and rescue phases. Data are expressed as the mean ± SEM. (**E**) Duration of nociceptive behavior (paw licking/flinching) following exposure to acetone for sham-treated (dashed line, *n* = 8) and SNI (solid line, *n* = 16) mice. SNI mice were treated with V1-cal (green, *n* = 8) or TAT_47–57_ (purple, *n* = 8) during the rescue phase. *P < 0.05 and **P < 0.01, SNI plus TAT_47–57_ versus sham; ^#^*P* < 0.05 and ^##^*P* < 0.01, SNI plus TAT_47–57_ versus SNI plus V1-cal. Significance was determined by 2-way RM ANOVA analysis with a mixed-effects model followed by Bonferroni’s post hoc test (**B**, **C**, and **E**) and unpaired, 2-tailed *t* test for the injury phase and 1-way ANOVA followed by Tukey’s post hoc test for the rescue phase (**D**).

Therefore, we further questioned whether delivering the V1-cal peptide through an osmotic pump could rescue the behavioral changes in the SNI model in WT mice. Two weeks after surgery, the mice subjected to SNI were randomly chosen to receive, via an osmotic pump, TAT_47–57_ vehicle or V1-cal (Figure 9A). We noted few changes in thermal latency in the SNI rodents relative to the sham-treated rodents during the injury or rescue phase (Figure 9B). The rodents subjected to SNI had a significantly lower mechanical withdrawal threshold during the injury phase than did the rodents subjected to the sham procedure (Figure 9C; 0.13 ± 0.04 vs. 1.32 ± 0.15 g, respectively, *P* = 0.0003). Further, during the rescue phase, the subset of SNI rodents given V1-cal had a substantial reversal of the mechanical withdrawal threshold compared with the rodents treated with TAT_47–57_ (Figure 9C; 1.25 ± 0.25 vs. 0.30 ± 0.03 g, respectively, *P* = 0.0144). This reversal was comparable to the mechanical threshold for the sham-treated rodents (Figure 9C; 1.14 ± 0.13 g). When measuring the percentage of hypersensitivity relative to the uninjured paw, the SNI rodents exhibited a substantial increase in hypersensitivity relative to the sham-treated rodents (Figure 9D; 87.1% ± 2.5% vs. 3.3% ± 1.8%, respectively, *P* < 0.0001). During the rescue phase, the rodents treated with V1-cal had reduced hypersensitivity, which markedly improved when compared with the TAT_47–57_–treated rodents (Figure 9D, 16.4% ± 3.0% vs. 76.8% ± 2.7 %, respectively, *P* < 0.0001). As cold allodynia also has a robust response after SNI, we repeated these studies in an additional group of mice by testing their response to acetone. V1-cal administration, as opposed to TAT_47–57_, also rescued the licking and flinching behavior observed with acetone treatment (Figure 9E).

## Discussion

Here, we identified a human TRPV1 genetic variant occurring just past the evolutionarily conserved TRP box (residues 696–701) within the C-terminal TRP domain that alters the TRPV1-mediated response to noxious insults (10, 26). The CRISPR/Cas9-edited TRPV1^K710N^-knockin mice provide evidence that this TRPV1 variant reduces, yet does not completely abolish, TRPV1-mediated responses without changes in the thermal response. Additionally, the reduced nociceptive response in TRPV1^K710N^ mice did not aggravate cellular injury but protected cells against noxious insults. As such, this study demonstrates that a single amino acid variant in TRPV1 changed nociceptive behavior in rodents. Based on these findings, we demonstrated that a TRPV1 peptide targeting the K710 region markedly reduced acute pain in addition to rescuing nociceptive behavior after nerve injury.

Since the capsaicin receptor was cloned, the TRPV1 channel is recognized as a molecular integrator of noxious insults (27). Caterina et al. reported that TRPV1-knockout mice are resistant to capsaicin-evoked behavior and have a reduced response to noxious heat (28). Our results for the TRPV1^K710N^-knockin mice show that the TRPV1 channel can be modified by CRISPR/Cas9 to separate capsaicin-evoked behavior from the thermal response. In turn, this effect can be mimicked by a peptide targeting this TRPV1 region. Leaving the TRPV1-mediated thermal response intact is important, as an increased thermal threshold could result in people inadvertently burning themselves (29). Additionally, TRPV1 antagonists such as AMG9810 can cause hyperthermia (30), which has hindered the development of an analgesic targeting TRPV1 (31). Here, V1-cal limited TRPV1-mediated pain responses without causing hyperthermia. The results of our study suggest that a peptide-based approach to modulate the TRPV1 receptor avoids the complications seen with TRPV1 gene knockout or with administration of TRPV1 antagonists.

We also demonstrate that TRPV1^K710N^-knockin mice had a reduced intensity of CGRP within DRG neurons. Prior literature reported that CGRP release contributes to neurogenic inflammation (32) and that capsaicin can increase CGRP levels in DRGs after injection (25). Furthermore, isolated mesenteric arteries of TRPV1-knockout mice release less CGRP with electric field stimulation (33). Our findings in TRPV1^K710N^-knockin mice, including reduced paw thickness and blood-flow response after capsaicin, indicate that TRPV1^K710N^-knockin mice have a weaker response to capsaicin-induced neurogenetic inflammation. Furthermore, in isolated vessels, TRPV1^K710N^-knockin mice also had a decreased vasodilatory response to capsaicin. Since TRPV1-expressing sensory neurons induce neurogenic inflammation via the release of neuropeptides including SP and CGRP, leading to vasodilation and tissue edema (34), we also found that a peptide targeting this TRPV1 region, V1-cal, could also reduce these markers of neurogenic inflammation in WT TRPV1 mice. Together, these findings show that this TRPV1 missense variant leads to a reduced response in neurogenic inflammation.

Initial studies using TRPV1-knockout rodents show that TRPV1 was not involved in the mechanism of nerve injury–induced hypersensitivity (28, 35). However, TRPV1 knock-down by siRNA or pharmacological inhibition reverses the mechanical hypersensitivity in a spinal nerve ligation mice model (36). This is opposed to the TRPV1-knockout rodents tested in the same study, which exhibited similar behaviors in mechanical hypersensitivity relative to WT rodents (36). In addition, the hypersensitivity caused by chronic constriction injury of the sciatic nerve could also be rescued by administration of siRNA targeting TRPV1 administered 7 days after injury or by the TRPV1 antagonist AMG-517 (37, 38). Prior studies indicate that nerve injury–induced mechanical hypersensitivity is dependent on IB4^+^ neurons and independent of CGRP (39–42). In this context, in the TRPV1^K710N^ mice, the reduced baseline expression of TRPV1 in IB4^+^ nonpeptidergic neurons and attenuated nocifensive behaviors during nerve injury, could be attributed to the lower population of IB4^+^TRPV1^+^ DRG neurons relative to that in WT TRPV1 mice. Given that the TRPV1 receptor remained functional in TRPV1^K710N^ mice, this study provides further insight into the differences in results when using mice with knockout of TRPV1 as opposed to knockdown or pharmacological inhibition of TRPV1. We also found that V1-cal attenuated cold allodynia in mice subjected to SNI, which could be due to direct regulation by TRPV1 of the response to TRPM8-mediated cold allodynia (43).

Acute or persistent pain behaviors are usually triggered by tissue or cellular injury, and the ability to detect noxious insults is essential to protect from further injury. As TRPV1-knockout mice have exacerbated post-ischemia inflammation and injury (44, 45), it is important to highlight that TRPV1^K710N^ mice could have reduced post-insult pain behaviors without increased cellular injury. In support of this idea, the TRPV1^K710N^ cardiomyocytes were resistant to both H_2_O_2_- and hypoxia-induced cellular injury in addition to cerebral ischemia-reperfusion injury. Rapid production of ATP from glycolysis is crucial for ischemic organs to meet energy demands and neuronal activity under stress (46, 47). We found that the glycolytic capacity in primary adult cardiomyocytes from TRPV1^K710N^ mice was enhanced under stress. The enhancement of glycolysis during ischemia could reduce ischemic damage and improve cardiac function with reperfusion (48). Therefore, the enhanced glycolytic capacity of TRPV1^K710N^ cardiomyocytes might be beneficial by effectively providing energy and thus protecting the cells against stress or ischemic insult.

The K710 site of TRPV1 is important in the regulation of TRPV1 function. Through site-directed mutagenesis, previous studies identified that K710 is a critical amino acid involving phosphatidylinositol 4,5-bisphosphate–mediated (PIP2-mediated) TRPV1 activation (49). Moreover, the K710 site can be directly bound by LPA, a phospholipid that is important in the generation and maintenance of pain (19, 50). K710 is a positively charged residue within the C-terminal TRP domain of the TRPV1 channel, and the charged amino acid is important in stabilizing the α-helix (51). We found that the K710N mutation in rat TRPV1, which introduces a neutrally charged amino acid residue, markedly reduced the calcium influx evoked by capsaicin, indicating the importance of K710 in capsaicin-induced TRPV1 gating. We further verified the in vitro data in the CRISPR/Cas9-edited TRPV1^K710N^-knockin mice. A previous study revealed that substitution of K710 with a neutrally or negatively charged amino acid causes a marked decrease in the TRPV1 current evoked by LPA in HEK293 cells but does not alter the response to capsaicin (19). Consistent with those data, our results show that TRPV1^K710N^ mice were resistant to acute pain induced by the LPA analog Brp-LPA, which can induce pain by direct activation of TRPV1 independent of LPA receptors (19). However, unlike the previous study, responses to capsaicin were also impaired because of the K710N mutation in cells and rodents. These findings suggest that capsaicin-induced TRPV1 activation is determined not only by the previously described critical binding site of Y511 located in the region linking the second and third transmembrane segments (13), but also by the K710 residue in the C-terminal TRP domain that contributes to channel gating.

Although CRISPR/Cas9-based technology allows researchers to effectively edit single or multiple genes in vitro and in vivo, there are still concerns regarding safety and ethics such as off-target effects or undesirable side effects. In this study, we did not perform whole-genome sequencing on TRPV1^K710N^ mice to rule out the possibility of off-target mutations. However, we used a pronuclear microinjection method to directly deliver the Cas9 protein and gRNA, which could largely reduce the off-target effects (52). Moreover, off-target mutations are rare in Cas9-edited mice (53), and we also initially backcrossed TRPV1^K710N^ mice with WT C57BL/6 mice to further remove potential interchromosomal off-target mutations. Although the role of TRPV1 in pain transmission and thermoregulation is well established, several reports indicate that TRPV1 is also broadly distributed within the brain and potentially involved in memory and motor function (54–56). Considered within the context of our testing, TRPV1^K710N^ mice had a normal appearance, fertility, and motor function, suggesting that the K710N genetic variant did not have a profound impact on brain function. As our behavioral assays were mainly reflex or spontaneous assays, our results are potentially limited, as we did not assess operant or pain-affected complex behaviors. Furthermore, we only assessed hypersensitivity, and not spontaneous pain or nonreflexive withdrawal, in our nerve injury studies. We also used Fura-2 changes in calcium influx to assess TRPV1 function, however, we did not use patch clamping to investigate how the K710N TRPV1 variant affects TRPV1 function. A prior report using patch-clamped endothelial cells describes how acute administration of V1-cal can block 12-HETE–induced activation of TRPV1 (15). We also did not use TRPV1-knockout mice to determine whether there are off-target effects of the V1-cal peptide for a SNI model. However, a prior study using TRPV1-knockout rats in a cardiovascular model showed that V1-cal selectively targets TRPV1 (23). Furthermore, although the K710N variant is reported at a low frequency in humans (1.645 × 10^–5^ frequency), according to the gnomAD (ID: 17-3475514-C-A), there is no pathophysiology related to this point mutation.

In summary, we generated a CRISPR/Cas9-edited TRPV1^K710N^ mouse based on a human missense variant within the TRPV1 TRP domain that results in human TRPV1 having avian-like TRPV1 qualities. The avian-like TRPV1^K710N^ mice provide evidence that the K710 site in the C-terminus of mammalian TRPV1 is critical for channel gating and nociception. Our data suggest that mitigating, yet not eliminating, the TRPV1-mediated response to noxious stimuli can limit pain behaviors while reducing cellular injury. A peptide targeting the region spanning the K710 residue further provides a non-opioid therapeutic by targeting a genetically divergent region between avian and mammalian species to limit pain transmission.

## Methods

Additional details on methods are provided in the Supplemental Methods.

### gnomAD database analysis

The single-nucleotide variants of the *TRPV1* transcript (ENSG00000196689.6) were accessed via the gnomAD, version 2.1.1. Synonymous single-nucleotide variants within the 3′- and 5′-UTRs or introns were filtered to include only missense variants and predicted loss-of -function variants for analysis. The human TRPV1 missense variants and predicted loss-of -function variants were then compared with genetically divergent residues in avian TRPV1 sequences to identify variants that cause a divergence between human and avian TRPV1.

### In silico structural analysis

To further investigate these identified variants, we performed in an silico analysis based on the published crystal structure of closed-state rat TRPV1 (PDB ID: 3J5P) (10). Furthermore, we focused on the TRP domain, and the substitution of Thr>Ile 708 (T708I) or Lys>Asn 710 (K710N) was individually introduced into the TRPV1 monomer structure model using the UCSF Chimera program (57). The rotamer with the highest probability was applied followed by 100 cycles of energy minimization. PyMOL version 2.0 (Molecular Graphics System, Schrödinger) was used to visualize the resulting structure models and check the loss or gain of polar interactions between neighboring residues located adjacent to the mutation.

### Rodent studies

All animal experiments were performed in accordance with Animal Research: Reporting of In Vivo Experiments (ARRIVE) guidelines (58). Animals were housed under standard temperature (21°C ± 2°C) and humidity (55% ± 5%) conditions with ad libitum access to food and water and were kept on a 12-hour light/12-hour dark cycle. The WT TRPV1 and TRPV1^K710N^ animals were allocated to experimental groups by age matching, and researchers were blinded to the genotypes of the mice when conducting animal experiments and analyzing the data.

### Generation of CRISPR/Cas9-edited TRPV1K710N-knockin mice

CRISPR/Cas9 gene-editing procedures were performed at the Transgenic, Knockout and Tumor Model Center (TKTC) at Stanford University. TRPV1^K710N^ mice were generated by introducing a mixture of gRNA (15 ng/L), donor oligonucleotide DNA (5 ng/μL), and Cas9 protein (30 ng/μL) by pronuclear microinjection into C57BL/6 mouse zygotes. Over 100 zygotes were injected and implanted into the oviducts of surrogate mothers. A total of 16 pups were born, and genomic DNA was extracted from tail biopsies followed by PCR amplification using a specific primer set to identify founders (F0). The PCR products were subjected to a T7EI cleavage assay to confirm Cas9-mediated, on-target cleavage of TRPV1. DNA-Seq analysis was used to further determine the mouse genotype. The correctly integrated mutant F0 mice were further backcrossed with WT C57BL/6J mice to produce offspring (F1) followed by intercrossing for 2 additional generations to obtain TRPV1^K710N^ homozygotes. Sequence information on gRNA, ssODNs, and primers are provided in Supplemental Table 2.

### Capsaicin-laced food test

Male and female WT TRPV1 and TRPV1^K710N^ C57BL/6 mice at 10–20 weeks of age were used. On the day of testing, 5 mice from each group were placed in a clean mouse cage (no bedding inside). The same amount (10 g) of capsaicin-free bird food (Deck, Porch N’ Patio, Wild Delight) was placed on the cage bottom, and the behavior responses were recorded by a video. Seven days after this test, the same mice were administered capsaicin-laced bird food (10 g, Sizzle N’ Heat, Wild Delight). For a 10-minute period, defensive withdrawal behavior, defined as at least 3 paws climbing on the cage wall or jumping, was counted by an observer blinded to the genotype of the mice.

### Behavioral testing

The behavioral experiments were conducted between 9 am and 4 pm and were in accordance with the ethics guidelines of the International Association for the Study of Pain (59). Male and female WT TRPV1 and TRPV1^K710N^ mice, at 10–20 weeks of age, were acclimated to this environment for 15 minutes prior to starting the experiment. The doses of capsaicin and Brp-LPA used to trigger pain behaviors in this study were based on prior literature (19, 60). Capsaicin (1.5 μg/paw in 20 μL, MilliporeSigma), Brp-LPA (4.1 μg/paw in 20 μL, Echelon Biosciences), or the corresponding vehicle (0.25% ethanol in saline or saline alone, respectively) was administered intraplantarly. Furthermore, the V1-cal peptide (1 μg/paw in 20 μL, GenScript) or TAT_47–57_ (1 μg/paw in 20 μL, Genscript) was administrated intraplantarly 15 minutes before capsaicin or Brp-LPA injection. Immediately after injection, nociceptive behavior (paw-licking and flinching) was quantified for 5 minutes immediately after injection (19, 61). Paw thickness was also measured 20 minutes after capsaicin injection using a digital caliper placed near the injection site. Each measurement was repeated 3 times. The observer was blinded to the mouse genotype.

### Laser Doppler blood flow measurement

Rodents were anesthetized with isoflurane (2%) and placed on a temperature-controlled heating pad to maintain body temperature within a normal range (37°C–38°C). Blood flow in the plantar skin was monitored by a laser Doppler Flowmeter (Perimed, assessed as AU) with a flexible fiber optic probe gently fixed to the paw injected with capsaicin (1.5 μg/paw in 20 μL) or vehicle (ethanol in saline, 20 μL). Baseline blood flow was measured for 10 minutes, and monitoring of blood flow was continued for 20 minutes after capsaicin injection.

### Temperature measurements

#### Rectal temperature.

To examine the effects of peptides and AMG9810 on temperature, V1-cal (1 mg/kg), TAT_47–57_ (1 mg/kg), AMG9810 (30 mg/kg, Alomone Labs, Israel), or the corresponding vehicle (saline for peptides and 5% ethanol in saline for AMG9810) was intraperitoneally injected. The doses of peptides and AMG9810 were based on prior literature (23, 62). The rectal temperature of the mice was measured at 10-minute intervals for a total of 90 minutes after the administration of drugs. The temperature was measured using a rectal thermal probe (diameter 1.5 mm) connected to a digital thermometer (Ruiwode), with insertion of the rectal probe to a depth of 1.5 cm for at least 10 seconds. The measurement was performed at a room temperature of 24.0°C.

#### Body temperature.

Mice were housed for the duration of the testing in a temperature-controlled animal housing facility monitored and maintained at a temperature of 22°C ± 0.4°C at Stanford University. A temperature probe (14 × 2.2 mm, 120 mg, IPTT-300 Temperature Transponder, Bio Medic Data Systems) was implanted subcutaneously dorsal and lateral to the L1–L4 region of the mouse, with a reported accuracy of ±0.6°C (63). After 3 days, body temperatures were measured in the morning and afternoon using a scanner (DAS-8007IUS Reader, Bio Medic Data Systems) that allowed for temperature measurements in conscious mice in home cages.

### Pressure myography

Third-order mesenteric arteries of WT TRPV1 or TRPV1^K710N^ mice were dissected under the microscope, and remaining fat was removed. Arteries were mounted on cannulas in a vessel chamber (Living Systems Instrumentation). The chamber was filled with calcium buffer, and a pressure of 80 mmHg was applied to each vessel. After the generation of myogenic tone, 10 nM (R)-phenylephrine hydrochloride (Phe) was added for preconstriction. After preconstriction, increasing concentrations of capsaicin (10^–9^ to 10^–4^ M) were added at 1-minute intervals, and the relaxation response was measured using digital video edge detection. In a separate series of experiments, WT TRPV1 arteries were perfused with 1 μM V1-cal or TAT_47–57_ for 3 minutes before increasing concentrations of capsaicin were given.

### Immunohistochemistry

Mice were euthanized and perfused intracardially with PBS followed by 4% buffered paraformaldehyde. Subsequently, the DRGs at the L4–L5 segments were removed and fixed in 4% buffered paraformaldehyde for 24 hours followed by immersion in 30% sucrose overnight. The tissue samples were embedded in OCT compound, and cryostat sections (10 μm) were prepared in a cryostat (NX50, Thermo Fisher Scientific) at –20°C. The sections were blocked and then incubated with the following primary antibodies: mouse anti-TRPV1 (75-254, NeuroMab) and rabbit anti-CGRP (14959, CST) or rabbit anti-CGRP (14959, CST) and guinea pig anti-SP (GP14110, Neuromics) at 4°C overnight. After incubation with primary antibodies, the sections were washed with PBS and then incubated with Alexa Fluor 488– or 594–conjugated secondary antibodies (Invitrogen, Thermo Fisher Scientific) at 37°C for 1 hour. For IB4 staining, DRG sections were incubated with isolectin B4 (BSI-B4), FITC conjugate (L2895, MilliporeSigma) at room temperature for 2 hours. Sections were washed in PBS, mounted in antifade mounting medium with DAPI (Invitrogen, Thermo Fisher Scientific), and imaged under a fluorescence microscope (Zeiss) followed by analysis of the fluorescence intensity with ImageJ, version 1.51s (NIH).

### Glycolysis stress test

A glycolysis stress test was conducted using the Seahorse extracellular flux analyzer (XFp Seahorse Bioscience) following the manufacturer’s instructions. Adult cardiac myocytes from WT TRPV1 or TRPV1^K710N^ mice were seeded in an 8-well, laminin-precoated miniplate at a density of 1,000 cells/well. After culturing overnight, the cells were washed twice using Seahorse XF Base Medium without glucose or pyruvate and supplemented with 2 mM glutamine (pH 7.4). Glucose (5.5 mM) was injected to induce a glycolytic response, oligomycin (2 μM) was injected to inhibit mitochondrial ATP production, and 2-DG (50 mM) was injected to inhibit glycolysis through competitive binding to glucose hexokinase. The real-time ECAR was directly measured to assess the key parameters of glycolytic function: glycolysis, glycolytic capacity, glycolytic reserve, and nonglycolytic acidification.

### SNI model

WT C57BL/6J or TRPV1^K710N^ male mice (10–16 weeks of age) were anesthetized with isoflurane (1%–3%) and placed on a heating pad to maintain body temperature at 37°C. The left hind leg was prepared and a longitudinal incision (~1cm) made in the skin proximal to the knee. Under a stereomicroscope, the muscle layer was blunt-dissected to reveal the common peroneal, tibial, and sural branches of the sciatic nerve. Leaving the sural nerve undisturbed, approximately 2 mm of the common peroneal and tibial nerves were transected and removed. The skin incision was then closed with a silk suture, and the mice were closely monitored during recovery. For the sham surgery, the common peroneal, tibial, and sural branches of the sciatic nerve were located but left undisturbed.

### Osmotic pump implantation and drug delivery

For Alzet osmotic pump implantation (volume 100 μL, Alzet model no. 1002), an incision was made at the nape of the mouse neck and the pump implanted immediately below the skin layer, which was closed with a silk suture for subcutaneous continuous delivery of the V1-cal peptide (1.0 mg/kg/d) or TAT_47–57_ (1.0 mg/kg/d) for 2 weeks from day 14 to day 28 after SNI surgery. The pumps were filled and then primed in 0.9% sterile saline at 37°C for approximately 24 hours before implantation.

### Mechanical, thermal, and cold nociception assessments

Mechanical, thermal, and cold nociceptive behaviors were assessed in mice subjected to SNI or a sham operation at baseline and at the post-SNI or sham operation and post-treatment stages of the protocol.

To assess mechanical nociception, mice were placed individually in a Plexiglass chamber on an elevated mesh screen stand and allowed to acclimate for a minimum of 10 minutes. The up-down technique was performed before (baseline) and after SNI or sham operation (64, 65). von Frey fibers ranging in force from 0.004 to 5.49 g were applied perpendicularly to the lateral surface of the hind paw until the paw was withdrawn. Once a response was observed, the next lower von Frey hair was applied. For each positive response (paw withdrawal), the next lesser filament was tested. For each negative response, the next higher filament was tested. The sequence of positive and negative responses was recorded and incorporated into an established curve-fitting algorithm to determine mechanical sensitivity (66).

To assess for changes in thermal nociception, the Hargreaves method was used to determine the hind paw withdrawal threshold. Mice were placed individually in Plexiglass chambers on a glass platform and allowed to acclimate for 10 minutes. The heat stimulus from a focused heat light source using a commercial Plantar Test Analgesia Meter (IITC Life Science) was applied to the ventral lateral surface of the hind paw with a light intensity of 30% determined in preliminary trials. The reaction time was measured in 0.01 second increments with a cutoff time of 15 seconds to prevent thermal damage to the paw region. A minimum of 30 seconds separated testing of the hind paws (67).

To assess acetone-induced cold nociception, 50 μL acetone solution was applied onto the lateral plantar surface of the paw using a blunt needle connected to a syringe, without touching the skin. The duration of the nociceptive response of paw licking and flinching was recorded for 60 seconds (68, 69).

### Statistics

On the basis of an a priori power analysis, we calculated that a minimum of 6 rodents were necessary to achieve at least a 20% minimal difference in behavioral responses and a minimum of 3 experimental replicates for nonbehavioral assays for a power of 80% and α = 0.05. Data are expressed as the mean ± SEM. Results were analyzed using GraphPad Prism 8.0 (GraphPad Software). An unpaired, 1- or 2-tailed *t* test was used for comparisons between 2 experimental groups. Comparisons among multiple groups were evaluated by 1- or 2-way ANOVA followed by a post hoc Tukey’s or Bonferroni’s test, or, when groups were uneven, a mixed-effects analysis model was used. Statistical significance was set at *P* < 0.05.

### Study approval

All animal studies conformed to the NIH’s *Guide for the Care and Use of Laboratory Animals* (National Academies Press, 2011). All animal studies were approved by the ethics committees of Stanford University, Anhui Medical University, Butantan Institute, and the University of Munich and were performed in accordance with the ethics standards laid down in the 1964 Declaration of Helsinki and its later amendments. All animal experiments were performed in accordance with Animal Research: Reporting of In Vivo Experiments (ARRIVE) guidelines (58). Behavioral experiments were conducted in accordance with the ethics guidelines of the International Association for the Study of Pain (59).

## Author contributions

ERG, SFH, SLM, NMW, and PS conceived and designed the experiments. SFH, VOZ, PS, YB, FR, LB, RCRH, BH, XY, MZ, BSN, ZFL, CW, SLM, and LJX performed the experiments and analyzed the data. SFH and ERG wrote the manuscript, which was revised by VOZ, PS, KJS, CMS, and YZ.

## Supplementary Material

Supplemental data

Supplemental video 1

Supplemental video 2

Supplemental video 3

Supplemental video 4

## Version 1 12/06/2022

In-Press Preview

## Version 2 02/01/2023

Electronic publication
