# Supplementary material for: A human TRPV1 genetic variant within the channel gating domain regulates pain sensitivity in rodents
Source: J Clin Invest. 2023 Feb 1;133(3):e163735. doi: 10.1172/JCI163735 (PMC9888391; doi:10.1172/JCI163735)
Supplement: Supplemental data [file jci-133-163735-s010.pdf]

Supplementary information for

A human TRPV1 genetic variant within the channel gating domain regulates pain sensitivity in rodents

Shufang He, Vanessa O. Zambelli, Pritam Sinharoy, Laura Brabenec, Yang Bian, Freeborn Rwere, Rafaela C.R. Hell, Beatriz Stein, Barbara Hung, Xuan Yu, Meng Zhao, Zhaofei Luo, Chao Wu, Lijun Xu, Katrin J. Svensson, Stacy L. McAllister, Creed M. Stary, Nana-Maria Wagner, Ye Zhang, Eric R. Gross*

*Correspondence author: Eric R. Gross

Email: ergross@stanford.edu

Materials and Methods

Phylogenetic tree construction

TRPV1 sequences of different species were obtained and exported as a FASTA file. The following amino acid sequences for TRPV1 were retrieved from GenBank: *Homo sapiens* (Human) NP_542436.2, *Rhinopithecus roxellana* (Monkey) XP_030778475.1, *Cavia porcellus* (Guinea pig) NP_001166123.1, *Rattus norvegicus* (Rat) NP_114188.1, *Mus musculus* (Mouse) NP_001001445.1, *Gallus gallus* (Bird) NP_989903.1, *Xenopus tropicalis* (Frog) NP_001243521.1 and *Danio rerio* (Zebra fish) NP_001119871.1. These sequences were aligned by ClustalW method and then a phylogenetic tree was constructed using a Neighbor-Joining (NJ) method by MEGA version 7.0.26 (1). The *Danio rerio* sequence was rooted as the outgroup, and the evolutionary distances were computed using the p-distance method based on the number of amino acid differences per site. The bootstrap test (500 replicates) in which the associated taxa clustered together was applied to compute the percentage of replicate trees. All positions with less than 50% site coverage were eliminated in evolutionary analyses.

Multiple sequence alignment

Multiple sequence alignment was performed by using Jalview 2.11.0 and the *Rattus norvegicus* (Rat) TRPV1 sequence was regarded as a reference sequence. A ClustalX color scheme was applied to label the residues (2), identifying different residues among mammalian and avian TRPV1sequence.

Site-directed mutagenesis

Site-directed mutagenesis was performed using QuikChange XL Site-Directed Mutagenesis Kit (Agilent, #200516) according to the manufacturer's instructions. The wild type rat TRPV1 clone on a pCMV6-Entry vector (OriGene Technologies Inc, RR213617) was used as a template. Primers for K710N, T708I, T708I/K710N mutants were designed using QuikChange Primer Design Program available online (www.agilent.com/genomics/qcpd). Polymerase chain reactions (PCR) were performed with the respective forward and reverse mutant primers using a PfuTurbo DNA polymerase followed by digesting the template DNA with Dpn I enzyme at a temperature of 37°C for 1hour. The Dpn I-treated DNA products were then transformed into XL10-Gold ultracompetent cells. The colonies of mutants were amplified, and the plasmid DNA was extracted for later use. The desired mutations were confirmed by DNA sequencing. All sequence information is presented in *SI Appendix* Table S1.

Cell culture and plasmid DNA transfection

H9C2 cells (ATCC® CRL-1446™) were cultured in Dulbecco modified Eagle medium (DMEM, Corning) supplemented with 10% fetal bovine serum (FBS, Gemini), 100 IU/ml penicillin, and 100 μg/ml streptomycin, maintained at 37 °C in a 5% CO_2 humidified incubator. Cells grown into ~70% confluence were transfected with the wild type or mutant constructs using Lipofectamine 2000 (Invitrogen) following the manufacturer's protocol. After 24 hours of transfection, the cells were seeded on polylysine pre-coated coverslips and cultured further for 24 hours.

Single cell calcium measurements

Calcium imaging was performed as previously described (3). Briefly, H9C2 cells or DRG neurons grown on glass coverslips were loaded with the 5μM Fura-2 AM (Thermo Fisher Scientific) in Hank's balanced salt solution (HBSS, with calcium/magnesium, no phenol red, Thermo Fisher Scientific) for 40 min at 37 °C. After dye loading, the cells were washed twice followed by 10 min of de-esterification. The coverslip was then transferred to a recording chamber mounted on the stage of a Zeiss inverted fluorescence microscope and continuously perfused with HBSS at a flow rate of 2 mL/min at room temperature (21-25°C). Calcium signal measurements were simultaneously recorded on individual cells using the fluorescence imaging system and EasyRatioPro software (Horiba) equipped with a galvanometer-based Random Access Monochromator (RAM) that can switch between any wavelength in 2 milliseconds (Horiba) and a Hamamatsu C11440 digital camera (Hamamatsu). Calcium measurement performed in Anhui Medical University utilized a calcium imaging system from Molecular Devices equipped with DeltaRAM-X, EMCCD and a MetaFluor software. Images and real-time calcium tracing data were acquired using an alternating excitation wavelength protocol (340, 380 nm/20 Hz) and emission wavelength of 510 nm. The normalized Fura-2 AM ratio 340/380 (subtract baseline ratio), the maximal change in Fura-2 ratio (peak ratio- baseline ratio), and the area under curve (AUC) of intracellular calcium response were calculated.

Rotarod test

Motor function was assessed with the accelerating rotarod system (SD Instruments). The experiments were conducted between 9 am and 4 pm. Wild type and TRPV1^{K710N} mice at 10-20 weeks of age, both male and female, were trained for walking on the 2.75 cm diameter rod rotating for three consecutive days and the mice were required to stay on the rod rotating at 5 rpm over 60 sec before proceeding to testing. On the testing day, mice were placed on the rod and the apparatus was set to accelerate from 4 to 40 rpm in 300 sec. The latency to fall from the rotating rod and the speed at fall were recorded, with a cut-off time of 600 sec. Procedures were repeated three times each day for each mouse separated by 15min intervals.

Tail immersion test

Wild type and $TRPV1^{K710N}$ male mice at 10-20 weeks of age, both male and female, were subjected to tail-immersion assay to test the nociceptive response to noxious heat. The animals in home cage were placed in testing room for at least 1hr before testing. During the test, mice were gently immobilized while allowed free tail movement. The tip of the tail (one-third of the length) is immersed in a water bath maintained at 48 °C or 55°C. The latency to a nociceptive response (withdrawal of the tail) is measured. Each mouse is given 3 trials with an intra-trial interval of 10 min. To prevent acute tissue damage, the tail is removed from the bath immediately after a nociceptive response or upon reaching a cut-off time of 30s.

Isolation of adult mouse primary DRG neurons

Male wild type TRPV1 and $TRPV1^{K710N}$ mice (10-16 weeks of age) were sacrificed under isoflurane anesthesia, and the DRGs from the cervical, thoracic, lumbar and sacral levels were aseptically removed and collected in HBSS (no calcium/magnesium, no phenol red, Thermo Fisher Scientific). Next, the DRGs were digested in 0.5% collagenase IA (Sigma) in HBSS at 37ºC for 1 hour. The cells were then incubated for 15 min in HBSS containing 0.1% trypsin (Sigma), washed in 2.5% trypsin inhibitor (Sigma) and then triturated using a thin fire polished pipette in DMEM, containing penicillin/streptomycin (1:100, Gibco) and 10% heat inactivated FBS (Sigma-Aldrich). Next, the cells were subjected to density gradient centrifugation at 200g for 1 min. The resulting pellet was resuspended in 10% FBS-DMEM, and the cell suspension was filtered through a cell strainer (75 μm, Becton Dickinson Labware). The DRG cells were then seeded onto Matrigel-coated glass coverslips and cultured in a humidified incubator at 37 °C with 5% CO_2. Calcium imaging were performed after 2 days in culture using Fura-2AM as calcium dye as described above.

Isolation of adult mouse primary cardiac myocytes

Male wild type TRPV1 and $TRPV1^{K710N}$ mice (10-16 weeks of age) were anesthetized under 2% isoflurane in 2L/min oxygen by vaporizer. A tail pinch was used to confirm the rodent had no response to surgical stimuli. Adult mouse ventricular cardiac myocytes were isolated and cultured as described before with modifications (4). In brief, mice under isoflurane anesthesia were subjected to a thoracotomy and hearts were rapidly excised then placed into a dish containing cold perfusion buffer (112 NaCl, 5.4 KCl, 1 $MgCl_2$, 9 Na_2HPO_4, 5.5 D-glucose, 10 HEPES, 30 taurine, 2 DL-carnitine, 2 creatine, 10 BDM, in mM, pH 7.3). After the aorta was cannulated, the heart was mounted on a Langendorff apparatus and perfused with the perfusion buffer at 37°C. After perfusion for 4 min at a flow rate of 4 ml/min, a digestion buffer containing collagenase type II (250 IU/ml, Worthington Biochemical), 20 μM $CaCl_2$ and

0.1% BSA were perfused for an additional 15 min until the heart became slightly pale and flaccid. To stop digestion, the left ventricles were removed, minced, then triturated in a wash buffer containing 1% BSA and 20 μM $CaCl_2$. The resulting cell suspensions were washed, and calcium was gradually reintroduced to a final concentration of 1.2mM. The final myocyte pellets were resuspended in a 4%FBS-Medium 199 (Thermo Fisher Scientific) containing 25 μM (-)-blebbistatin (Sigma-Aldrich) and seeded in a laminin (Sigma-Aldrich) pre-coated plate. After plating for 2 hours, the medium was changed to a 1% BSA-Medium 199 for maintaining culture.

Cellular oxidative stress

Cardiac myocytes were treated with 25μM H_2O_2 for 4 hours to induce cell oxidative injury. Further, cardiomyocytes were subjected to hypoxia by incubation in hypoxic buffer (125 mM NaCl, 8 mM KCl, 1.2 mM KH_2PO_4, 1.25 mM $MgSO_4$, 1.2 mM $CaCl_2$, 6.25 mM $NaHCO_3$, 20 mM 2-deoxyglucose, 5 mM Na-lactate, and 20 mM HEPES; pH 6.6) in hypoxic pouches (GasPak EZ, BD Biosciences) for 90 min at 37°C (5). The pouch creates an anaerobic environment where the oxygen level within the pouch is less than 0.1% (6). The cells were then removed from the anaerobic gas pouches and were reoxygenated with 1% BSA-Medium 199 medium under normoxia for 4 h. Cells in the sham group were continuously cultured in the 1% BSA-Medium 199 medium under normoxia, though changed medium at the same time points.

Calcein AM/PI staining

Cell death in cardiac myocytes were assessed by the calcein AM and propidium iodide (PI) for simultaneous fluorescence staining of viable and dead cells. The cardiac myocytes were plated in laminin-coated Nunc™ Lab-Tek™ 8-well Chambered Coverglass (Thermo Fisher Scientific). After treatment, the cells were stained with 1 μM calcein-AM (Thermo Fisher Scientific) and 5 μM PI (Sigma-Aldrich) in PBS for 15 min. Following dye loading, the images of the cells were acquired immediately by using a Zeiss LSM 900 confocal laser scanning microscope, and at least five fields were randomly selected for each well. The average fluorescence intensity was assessed with Image J 1.51s (NIH). The fluorescence intensity of the control group cells was assigned as 100%.

Cell viability assay

Cell viability was measured by the MTT assay as described previously(7). Briefly, cardiomyocytes were homogeneously seeded in a 48-well plate at a density of 5×10^4/ml. After treatment, the cells were incubated in culture medium containing 50 μg/mL MTT for 2 h at 37 °C in a 5% CO_2 incubator. After removal of the MTT medium, the

formazan products were sufficiently dissolved in dimethyl sulfoxide (DMSO) and then detected by a Microplate Reader (BioTek Instruments). The absorbance at 570 nm of the control cells was considered to be 100%.

Model of focal cerebral ischemia

Transient focal ischemia was induced by the intraluminal suture method of middle cerebral artery occlusion (MCAO) as previously described (8). Wild type and TRPV1^{K710N} male mice were anesthetized under 2% isoflurane and 70% N_2O. Cerebral ischemia was achieved by 1 hour of MCAO with a silicone-coated 6-0 monofilament (Doccol Corp, Redlands, CA) followed by reperfusion. Rectal temperature was maintained at 37±0.5°C with a heating pad (Harvard Apparatus, Holliston, MA). Mice with no evidence of acute neurological deficit or with evidence of hemorrhage were excluded from analysis. After 24 hours of reperfusion, mice were deeply anesthetized with isoflurane and decapitated. Brains were removed and sectioned coronally with a rodent brain slicer matrix (Zivic Instruments, Pittsburgh, PA). Sections were incubated in 2% 2,3,5-triphenyletrazolium chloride (TTC) and infarction volume determined using 4 slices per mouse analyzed by a blinded observer and corrected for edema using Image J 1.51S (NIH) as described previously (8).

Supplemental figures:

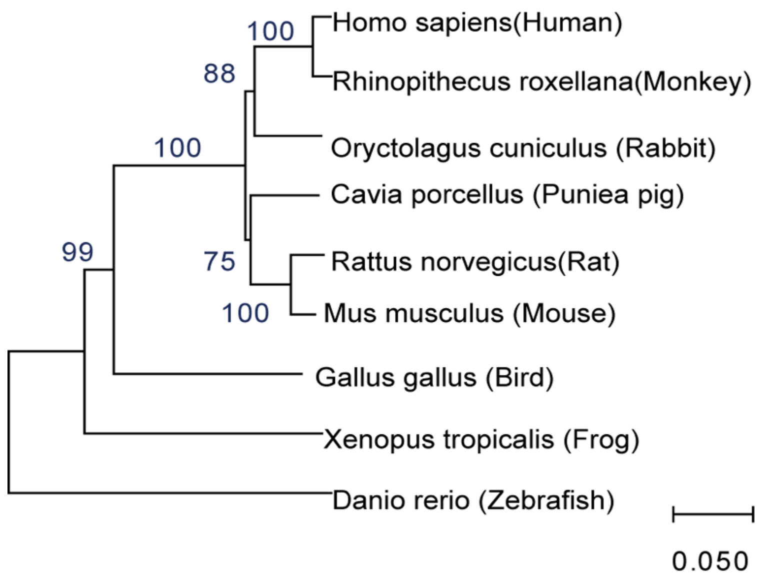

Figure S1 Neighbor-Joining phylogenetic tree for TRPV1. Percentage bootstrap values are shown on interior branches. The scale bar represents 0.05 amino acid substitutions per amino acid site.

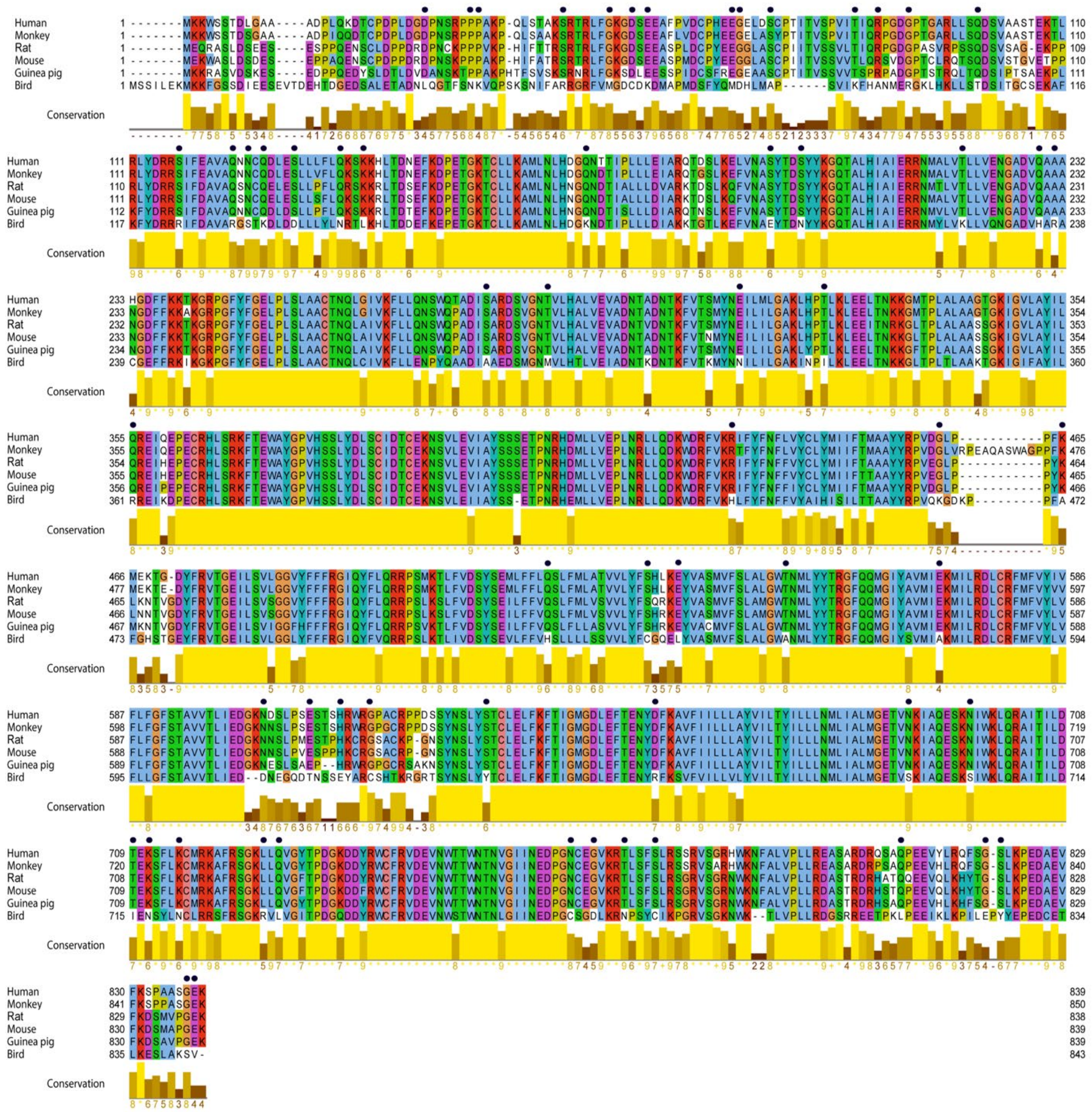

Figure S2 Multiple sequence alignment on mammalian and avian TRPV1 Sequence alignment on TRPV1 between mammals (human, monkey, rat, mouse and guinea pig) and bird was performed by Jalview program and the amino acids are colored by a ClustalX color scheme (blue: conserved hydrophobic amino acids, red: conserved positive charge amino acids, green: conserved polar amino acids, magenta: conserved negative charged animo acids, white: unconserved amino acids). Conservation is reflected by a numerical index with identities scoring highest, and amino acids with substitutions in the same physico-chemical class have next highest score. Conserved columns are indicated by *(score of 11), and columns with mutations where all properties are conserved are marked with a + (score of 10). Block dot (•) indicates the residue that identical among mammals but genetic divergent in birds.

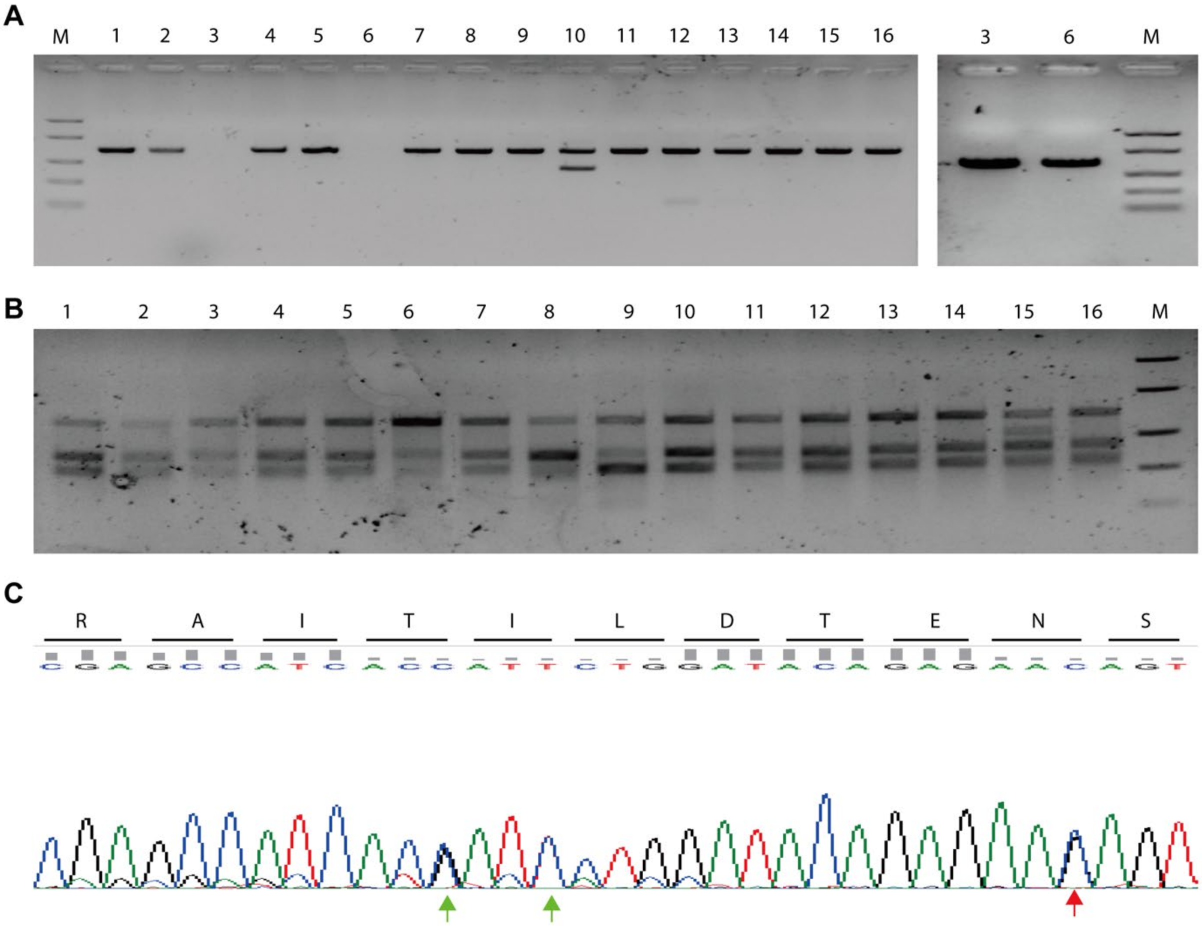

Figure S3 Verification of CRISPR/Cas9-edited TRPV1^{K710N} founder mice. (A) Agarose gel electrophoresis on PCR products of founder mice. (B) T7 endonuclease I (T7EI) cleavage of PCR products. (C) Representative DNA sequencing data for founder mice with designed mutation. Red arrow indicates the designed mutation, and the green arrows indicate silent mutations.

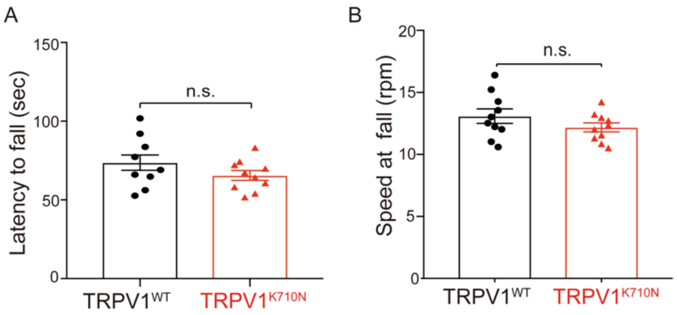

Figure S4. Rotarod testing of TRPV1^{WT} relative to TRPV1^{K710N} mice. (A) The latency time (sec) to fall from the rotating rod and (B) the speed (rpm) at fall from the rotating rod in wild type TRPV1 and TRPV1^{K710N} mice. n=10 in each group. Data are expressed as mean ± SEM. Unpaired two-tailed *t* test.

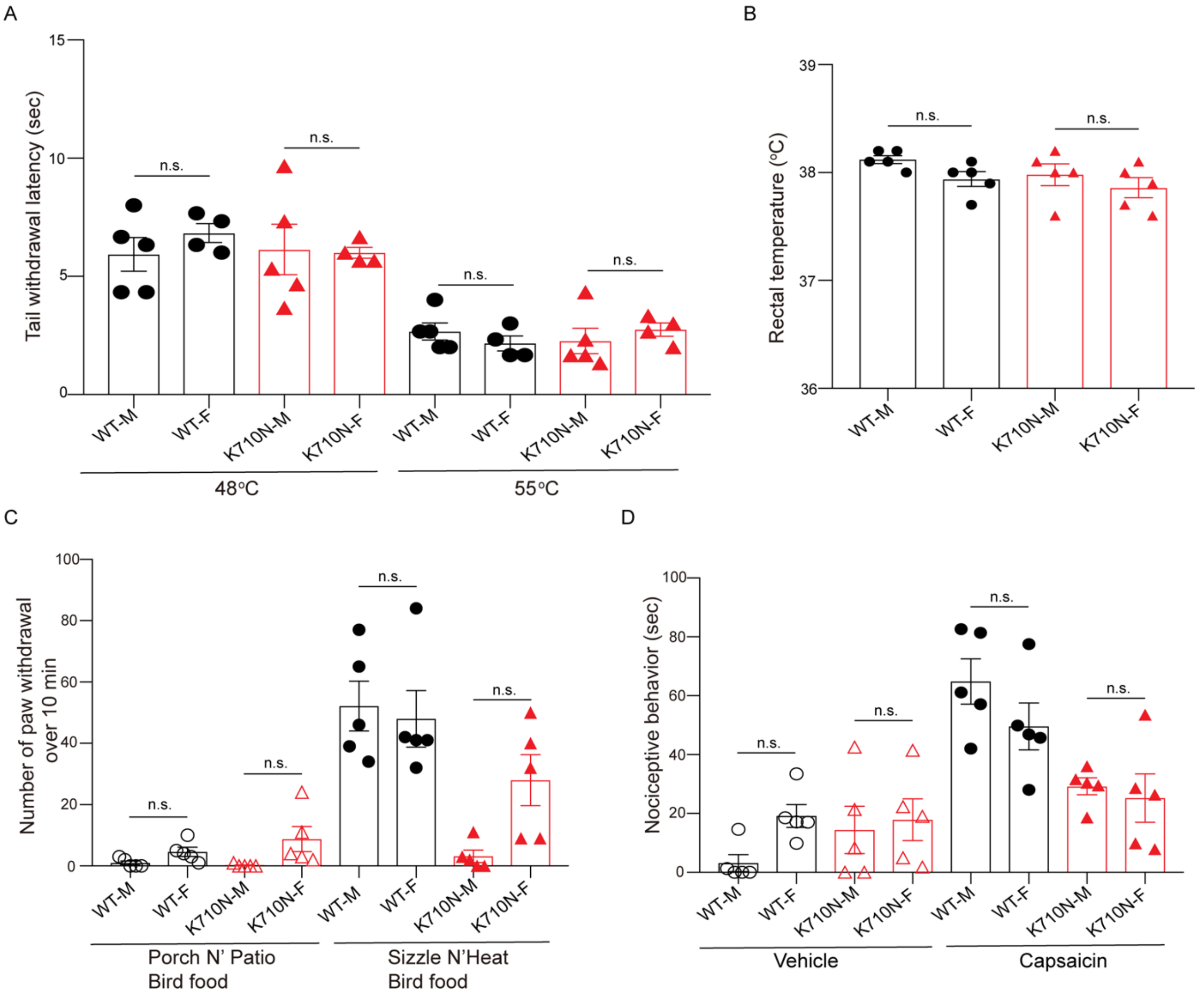

Figure S5 Sex difference in temperature testing and nocifensive behaviors in wild type TRPV1 and TRPV1K710N mice. (A) Tail withdrawal latency time (sec) in response to hot water at 48°C and 55°C in male and female wild type TRPV1 and TRPV1^{K710N} mice. (B) Body temperature detected by a rectal temperature probe in male and female wild type TRPV1 and TRPV1^{K710N} mice. n=4-5/group. (C) Paw withdrawal behavior for male and female wild type TRPV1 and TRPV1^{K710N} mice exposed to capsaicin-laced bird food (Sizzle N' Heat Bird food) or regular bird food (Porch N' Patio Bird food). (D) Nociceptive behavior by intraplantar capsaicin injection in male and female wild type TRPV1 and TRPV1^{K710N} mice. M, male mice; F, female mice. Data are expressed as mean ± SEM analyzed by one-way ANOVA followed by Tukey's *post hoc* test.

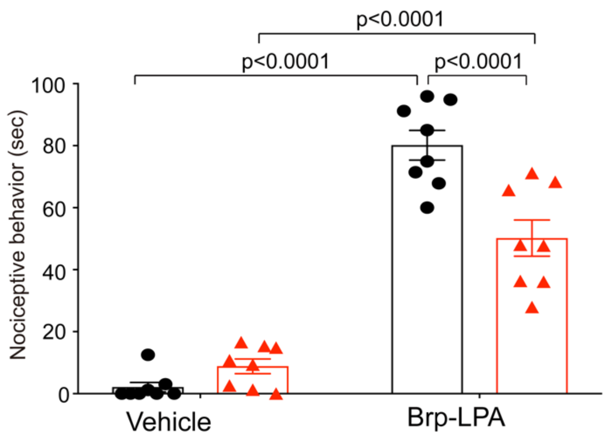

Figure S6. Acute nociceptive behavior induced by paw-injection of Brp-LPA. Pain behavior induced by paw-injection of Brp-LPA (8nmol/20µl) or vehicle (saline/20µl) in wild type TRPV1 (black dot) and TRPV1^{K710N} (red triangle) mice. n=8/group. Data are expressed as mean ± SEM analyzed by two-way ANOVA followed by Tukey's *post hoc* test.

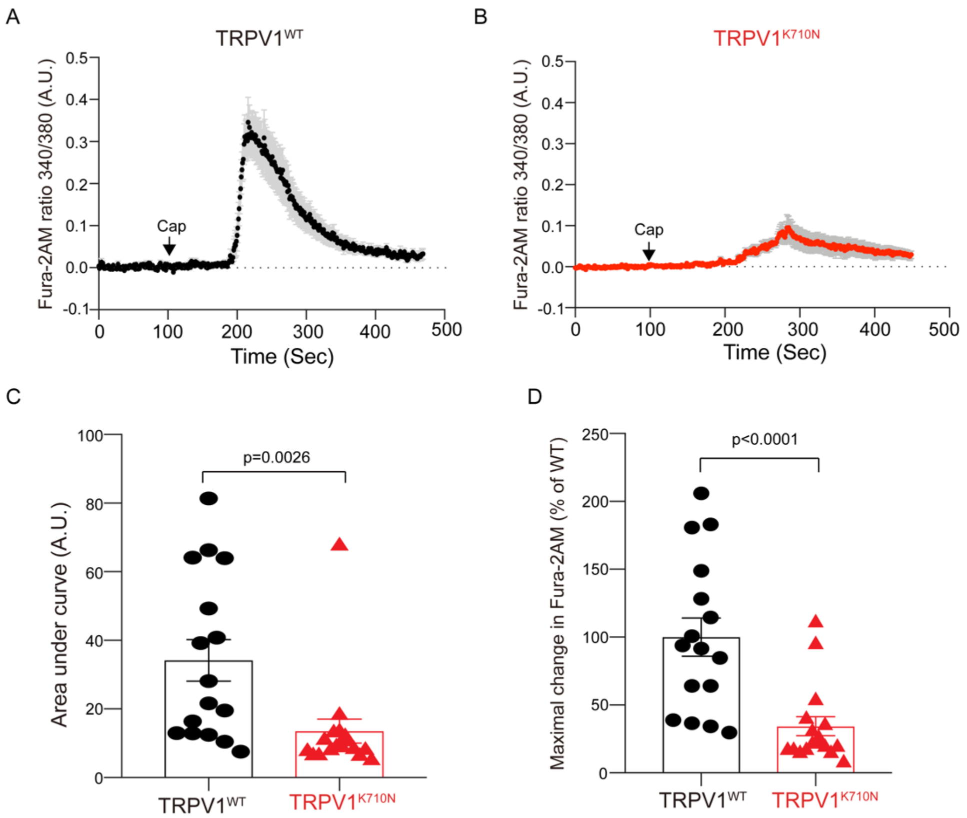

Figure S7 Calcium influx in response to capsaicin in DRG neurons from wild type TRPV1 and TRPV1^{K710N} mice (A, B) Fura-2 AM in response to capsaicin (Cap) of DRG cells from wild type TRPV1 and TRPV1^{K710N} mice presented as changes in ratio 340/380 nm. (C) The area under curve (AUC, total amount of calcium influx) and (D) the maximal rise (maximal change in Fura-2 ratio) of capsaicin-stimulated calcium influx. n=16 (wild type TRPV1) or 17 (TRPV1^{K710N}) with DRG cells from 3 independent experiments. Data are expressed as mean ± SEM and analyzed using a one-tailed *t* test (C, D).

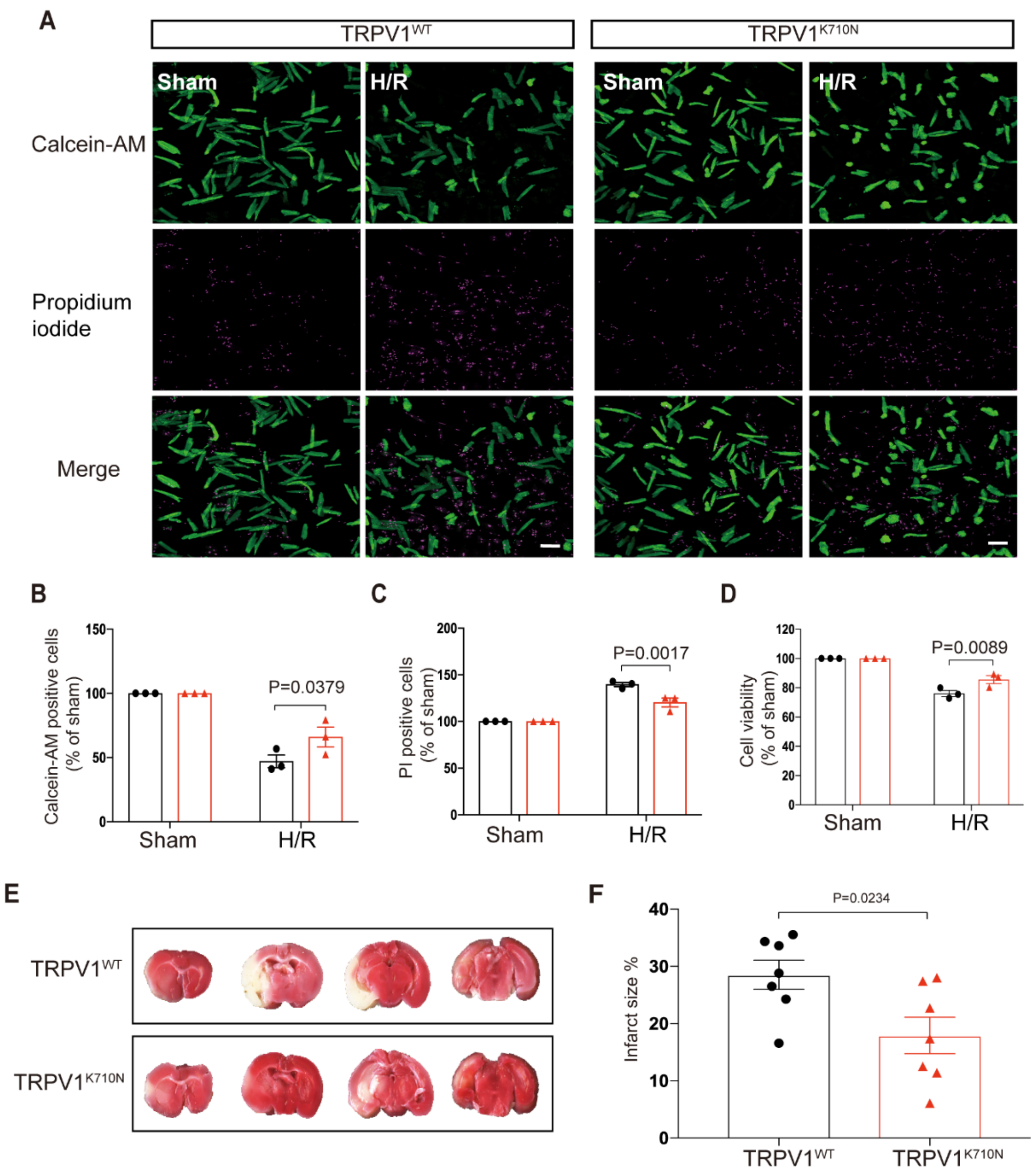

Figure S8 Ischemic injury in wild type TRPV1 and TRPV1^{K710N} mice. (A) Representative image of calcein AM (green) and PI (magenta) double stained cardiomyocytes after hypoxia/reoxygenation (H/R) or sham. Bar=100µm. **(**B) Calcein-AM stained viable cells or (C) PI stained dead cells were quantified as the average fluorescence intensity. n=3/group. **(**D) Cell viability measured by MTT. The value of sham cells in wild type (black) or TRPV1^{K710N} (red) cells was set as 100%, respectively. n=3/group. (E) Representative image showing TTC stained coronal sections following focal cerebral ischemia and reperfusion. (F) Quantification of infarct volume expressed as a percent of hemispheric volume. n=7/group. Data are expressed as mean ± SEM. In (B-D), two-way ANOVA followed by Bonferroni's *post hoc* test. In (F), Unpaired two-tailed *t* test.

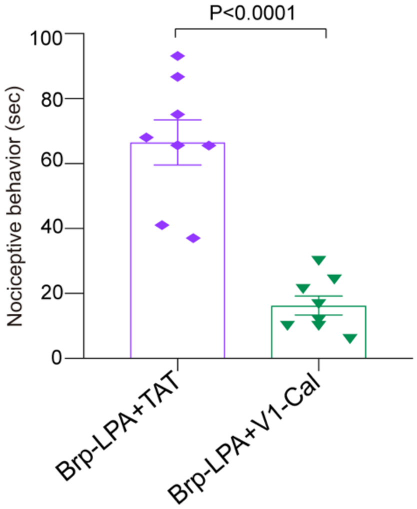

Figure S9 Acute nociceptive behavior induced by paw-injection of Brp-LPA. Pain behavior induced by paw-injection of Brp-LPA after V1-cal or TAT treatment in wild type TRPV1 mice. n=8/group. Data are expressed as mean ± SEM. Unpaired two-tailed *t* test.

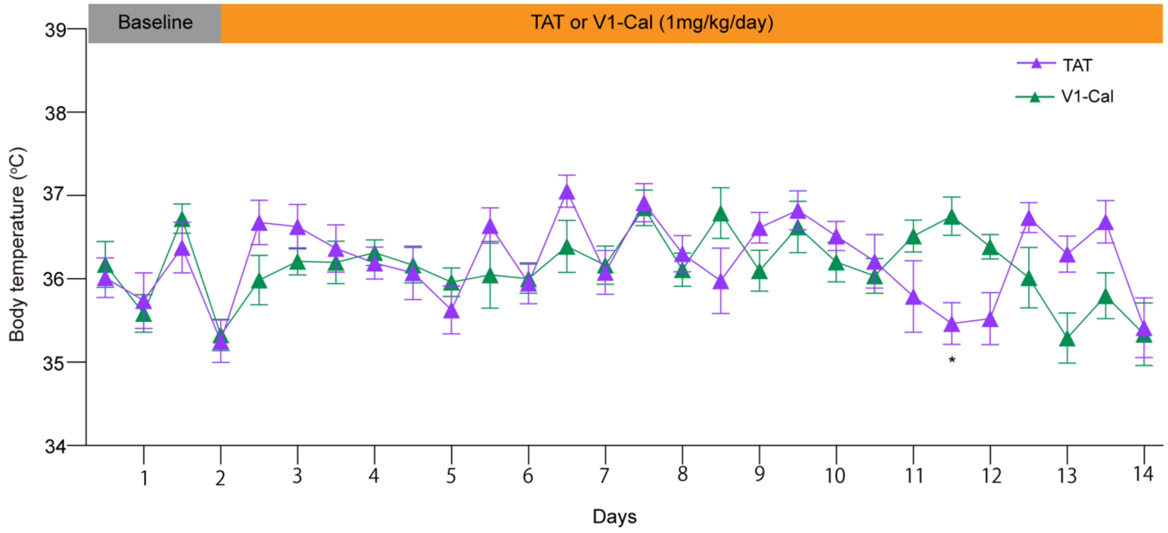

Figure S10 Body temperature measured by an implanted temperature probe. Body temperature was measured twice a day for 2 days before osmotic pumps implantation for baseline data, and continuously monitored over the subsequent 12 days with osmotic pumps infusion of TAT47-57 vehicle or V1-cal (1mg/kg/day). n=8/group. Data are expressed as mean ± SEM analyzed by two-way RM ANOVA followed by Bonferroni's *post hoc* test. * $p<0.05$ TAT vs. V1-Cal.

Tables:

Table S1. Primers sequence for rat TRPV1 site-mutagenesis

Primer name	Primer sequence (5'-3')
K710N Forward	CCATCCTGGATACAGAGAACAGCTTCCTGAAGTGC
K710N Reverse	GCACTTCAGGAAGCTGTTCTCTGTATCCAGGATGG
T708I Forward	GCCATCACCATCCTGGATATTGAGAAGAGCTTCCTGAAG
T708I Reverse	CTTCAGGAAGCTCTTCTCAATATCCAGGATGGTGATGGC
K710N/T708I Forward	AGAGAGCCATCACCATCCTGGATATTGAGAACAGCTTCCTGAAGT
K710N/T708I Reverse	AGAGAGCCATCACCATCCTGGATATTGAGAACAGCTTCCTGAAGTG
Sequencing primer	GCAAGCCAGGTAACTCTTAC

All primers were designed by using QuikChange Primer Design Program available online (www.agilent.com/genomics/qcpd), and synthesized by Integrated DNA Technologies (IDT) company.

Table S2. Sequence information for generating CRISPR/Cas9-edited mice

Name	Sequence (5’-3’)
gRNA1	ACTCTTCTCTGTATCCAGGA
gRNA2	GGAAACTCTTCTCTGTATCC
gRNA3	CCAGCGAGCCATCACCATCC
ssODN	TTGTAGTAGGCTGCCTCAGGGGCTGAAAGAGATTCTGGAGAGGGTGATC CAGGCTGGTGGCCTGTTTCCTTTTCCACCAGCGAGCCATCACGATTCTGG ATACAGAGAACAGTTTCCTGAAGTGCATGAGGAAGGCCTTCCGCTCC
PCR Forward primer	AAGGAATTCTGTCAGCATCTCATC
PCR Reverse primer	CCTTGAGAGTCCCTATTTCTATGTCTAG
Genotyping primer	AAGGAATTCTGTCAGCATCTCATC

The gRNAs were made from crRNA and tracrRNA by Integrated DNA Technologies (IDT) company. The ssODN was synthesized by IDT in which the red bases are designed mutation, the green bases are silent mutations. All primers were synthesized by IDT. gRNA, guide RNA; crRNA, CRISPR RNA; tracrRNA, trans-activating crRNA.

Supplemental Movie titles

Movie S1. Responses to capsaicin-laced food in wild type (A) and TRPV1^{K710N} mice (B).

Movie S2. Responses to capsaicin-free food in wild type (A) and TRPV1^{K710N} mice (B).

Movie S3. Nocifensive behavior after capsaicin paw-injection in wild type TRPV1 mouse.

Movie S4. Nocifensive behavior after capsaicin paw-injection in TRPV1 ^{K710N} mouse.

References:

1. Kumar S, et al. MEGA: Molecular Evolutionary Genetics Analysis software for microcomputers. *Comput Appl Biosci.* 1994;10(2):189-91.
2. Thompson JD, et al. The CLUSTAL_X windows interface: flexible strategies for multiple sequence alignment aided by quality analysis tools. *Nucleic Acids Res.* 1997;25(24):4876-82.
3. Sinharoy P, et al. TRPA1 and TRPV1 contribute to propofol-mediated antagonism of U46619-induced constriction in murine coronary arteries. *PloS one.* 2017;12(6):e0180106.
4. Li D, et al. Isolation and culture of adult mouse cardiomyocytes for cell signaling and in vitro cardiac hypertrophy. *Journal of visualized experiments : JoVE.* 2014(87):51357.
5. Kubli DA, et al. Bnip3 functions as a mitochondrial sensor of oxidative stress during myocardial ischemia and reperfusion. *Am J Physiol Heart Circ Physiol.* 2008;295(5):H2025-31.
6. Lu Y, et al. Transient Receptor Potential Ankyrin 1 Activation within the Cardiac Myocyte Limits Ischemia-reperfusion Injury in Rodents. *Anesthesiology.* 2016;125(6):1171-80.
7. Gomez LA, et al. Use of the MTT assay in adult ventricular cardiomyocytes to assess viability: effects of adenosine and potassium on cellular survival. *Journal of molecular and cellular cardiology.* 1997;29(4):1255-66.
8. Xiong X, et al. Increased brain injury and worsened neurological outcome in interleukin-4 knockout mice after transient focal cerebral ischemia. *Stroke.* 2011;42(7):2026-32.
